# Elucidation of Medusozoan (Jellyfish) Venom Constituent Activities Using Constellation Pharmacology

**DOI:** 10.3390/toxins16100447

**Published:** 2024-10-17

**Authors:** Angel A. Yanagihara, Matías L. Giglio, Kikiana Hurwitz, Raechel Kadler, Samuel S. Espino, Shrinivasan Raghuraman, Baldomero M. Olivera

**Affiliations:** 1 Pacific Biosciences Research Center, University of Hawaii at Manoa, Honolulu, HI 96822, USA; rkadler@hawaii.edu; 2 Department of Biology, University of Utah, Salt Lake City, UT 84115, USA; matias.giglio@utah.edu (M.L.G.); samuel.espino@utah.edu (S.S.E.);; 3 Faculty of Sciences, Brigham Young University Hawaii, Laie, HI 96762, USA; kikiana.hurwitz@byuh.edu

**Keywords:** venom, cubozoa, box jellyfish, *Physalia*, Fura-2 calcium imaging, constellation pharmacology, ion-channel modulator, dorsal root ganglia, neurons, glial cells

## Abstract

Within the phylum Cnidaria, sea anemones (class Anthozoa) express a rich diversity of ion-channel peptide modulators with biomedical applications, but corollary discoveries from jellyfish (subphylum Medusozoa) are lacking. To bridge this gap, bioactivities of previously unexplored proteinaceous and small molecular weight (~15 kDa to 5 kDa) venom components were assessed in a mouse dorsal root ganglia (DRG) high-content calcium-imaging assay, known as constellation pharmacology. While the addition of crude venom led to nonspecific cell death and Fura-2 signal leakage due to pore-forming activity, purified small molecular weight fractions of venom demonstrated three main, concentration-dependent and reversible effects on defined heterogeneous cell types found in the primary cultures of mouse DRG. These three phenotypic responses are herein referred to as phenotype A, B and C: excitatory amplification (A) or inhibition (B) of KCl-induced calcium signals, and test compound-induced disturbances to baseline calcium levels (C). Most notably, certain *Alatina alata* venom fractions showed phenotype A effects in all DRG neurons; *Physalia physalis* and *Chironex fleckeri* fractions predominantly showed phenotype B effects in small- and medium-diameter neurons. Finally, specific *Physalia physalis* and *Alatina alata* venom components induced direct excitatory responses (phenotype C) in glial cells. These findings demonstrate a diversity of neuroactive compounds in jellyfish venom potentially targeting a constellation of ion channels and ligand-gated receptors with broad physiological implications.

## 1. Introduction

Marine organisms, including sea anemones (phylum Cnidaria, subphylum Anthozoa), have proven to be rich sources of natural products and bioactive compounds with broad biomedical applications. In contrast, corollary discoveries from Medusozoa, the other cnidarian subphylum, which comprises four classes, Hydrozoa (e.g., *Physalia* sp.), Scyphozoa (radially symmetrical jellyfish), Cubozoa (box jellyfish), and Staurozoa (stalked jellyfish), are limited. That gap, as well as the fact that most medically significant stings are associated with a limited number of medusozoan species, including the species examined in this study, prompted this work. In parallel with the objective of natural product discovery, identification and characterization of the bioactive constituents comprising venoms of medusozoan (jellyfish) “stingers” causing human morbidity and mortality also accelerates understanding of the pathophysiology underlying medical sequelae following envenomation [1,2] essential to optimal sting management. To date, several classes of medusozoan venom components have been shown to contribute to tissue damage directly; these include porins, metalloproteinases and phospholipases [2,3,4,5]. In contrast, a systematic characterization of bioactivity of constituent venom peptides and small molecular weight medusozoan venom components (~15 kDa to 5 kDa) are lacking, despite the preponderance of evidence for their role in envenomation based upon transcriptomics analysis, e.g., [6,7,8].

Elucidating the diverse bioactivities of jellyfish venoms requires optimizing the recovery of venom constituents, as well as utilizing and developing appropriate high-content assays. For this reason, we previously rigorously compared extant methods of venom recovery to optimize bioactivity and yields [1]. Despite having markedly improved venom recovery and specific activity, the quantities and purity required for bioassay presented an impediment to progress. Specifically, the effects of venoms, or their components, have traditionally been studied through bioactivity-driven purification using whole animal, organ bath, and/or tissue culture cell assays. Fractionated lethal and bioactive toxins have been identified using these assays, which have facilitated the elucidation of pathophysiological mechanisms [9,10,11,12]. These approaches provide insights into envenomation pathophysiology but are impractical for in-depth and comprehensive analyses of complex venoms composed of hundreds of novel constituents. The advent of high throughput 96-well plate-based assays, such as Fluorescence Imaging Plate Reader (FLIPR), accelerated one arm of natural-product discovery, leading to the discovery of many molecular ligands for specific receptors using established cell lines [13].

Another approach to bioactive-constituent discovery has been electrophysiological recording in which the effects of test compounds on various voltage- and ligand-gated channels are determined. Electrophysiological methods, such as patch clamp or two-electrode voltage clamp, can measure small changes in the current flux or membrane potential of the cells at a millisecond scale. Through these methods, the effects of purified venom components can be assessed on individual molecular targets. However, these specific channel-recording techniques are time-consuming and purity- and concentration-dependent, while venom samples are typically complex mixtures with limited quantities [14], rendering the screening of hundreds of constituents over multiple concentrations and on multiple molecular targets unfeasible.

**Figure 1:**
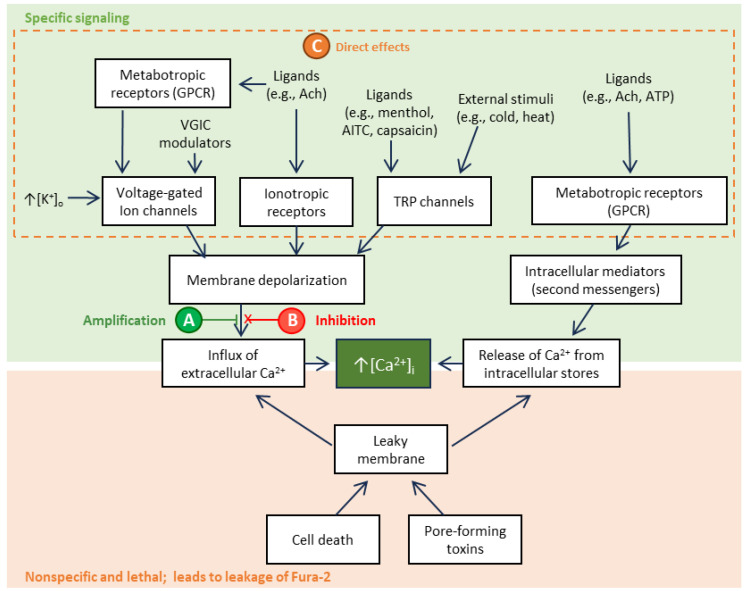
Biochemical signaling pathways that result in increased intracellular calcium concentration ([Ca^2+^]_i_). Different cell processes can lead to the influx of extracellular Ca^2+^ or the release of Ca^2+^ from the intracellular stores, both leading to increased [Ca^2+^]_i_. When assessing the effects of venom component toxins by constellation pharmacology using high extracellular potassium [K^+^] pulses (e.g., 25 mM KCl) as a depolarizing stimulus, three different effects could be observed according to the interaction of the venom components to one or more of the mechanistic pathways depicted here: amplification (phenotype A), which augments or prolongs subsequent membrane depolarization duration (e.g., potential mechanisms include blocking voltage-gated potassium channels or delayed inactivation of sodium channels); inhibition (phenotype B), which decreases or shortens the membrane depolarization duration (e.g., potential mechanisms include blocking voltage-gated sodium or calcium channels or activation of hyperpolarizing ion channels including GABA or glycine receptors); and direct effects (phenotype C) on voltage-gated ion channels and ligand-gated ion channels, including G-protein coupled receptors (GPCRs), causing a depolarizing elevation of cytosolic calcium levels. Finally, as with other calcium imaging-based assays, calcium ionophores and cytolytic events involving the loss of cell membrane integrity can result in a transient increase in the intracellular Ca^2+^, which is most often irreversible and leads to cell death with leakage of the Fura-2.

A novel breakthrough in venom bioactivity screening has been achieved based on a paradigm shift in experimental design strategy. The parameter measured in single-cell-based electrophysiological recordings is the membrane potential; changes in membrane potential lead to downstream changes in intracellular calcium (Ca^2+^) concentrations ([Ca^2+^]_i_) [15]. Additionally, ligand-coupled signaling can result in the release of Ca^2+^ from intracellular stores into the cytoplasm (Figure 1). Based on this phenomenon, intracellular Ca^2+^-binding dyes have been utilized as membrane potential “reporters”. The Ca^2+^ ion concentration reporting fluorophore, Fura-2, is a UV light-excitable ratiometric indicator for which the excitation maxima shifts from 380 nm to 340 nm (emission at 510 nm) upon coupling with Ca^2+^ ions. Thus, the ratio of 510 nm emission intensity after excitation at 340 nm compared with 380 nm reflects the [Ca^2+^]_i_ [14,16]. Hereafter, this ratiometric measurement will be referred to as “Fura-2 signal intensity”.

**Figure 2:**
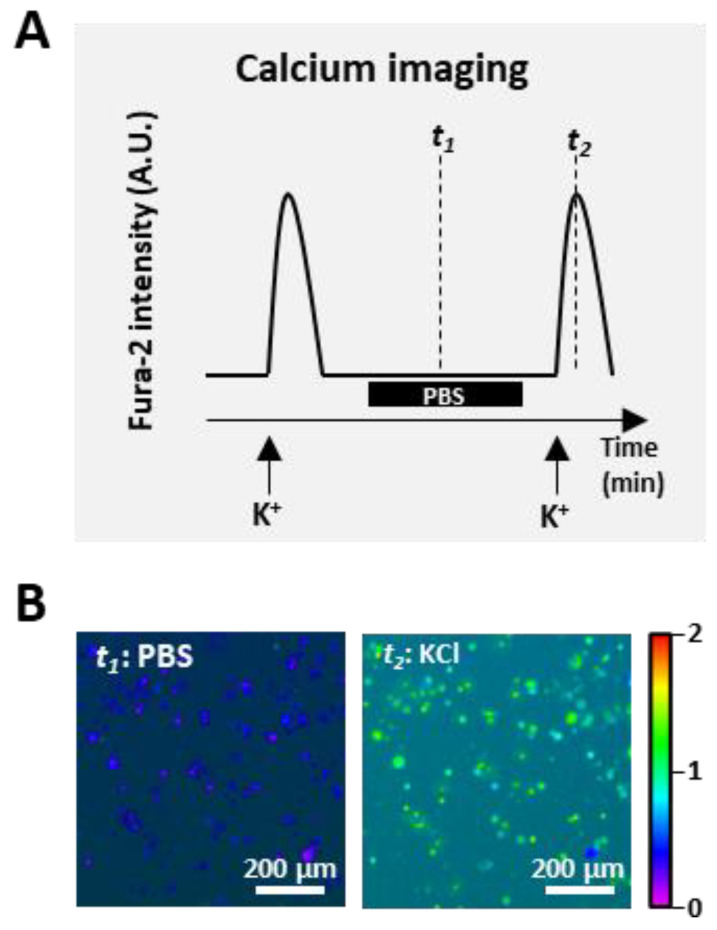
Output of the constellation pharmacology assay. **(A**) Representative tracing of two pulses of KCl with the application and incubation with phosphate-buffered saline (PBS) as a control. The application of extracellular KCl causes membrane depolarization, leading to an elevation of intracellular calcium and thus an increase in the Fura-2 signal. Note that the signal peak heights are reproducible and reversible; (**B**) Representative images of dorsal root ganglia (DRG) cells indicating Fura-2 intensity at the time of PBS incubation (*t*_1_) and during the application of KCl (*t*_2_) from A are shown.

Calcium imaging-coupled bioactivity assays have thus revolutionized traditional screening approaches. Rather than assessing the responses of a single cell, diverse cell types can be monitored with the stable fluorophore Fura-2 to allow simultaneous and temporal imaging of thousands of cells in a HEPES based buffer (“observation solution” Section 5.4) within the test well chamber. This approach is used for a novel discovery platform called constellation pharmacology [17,18]. Figure 2 shows responses to “pulse-chase” applications of extracellular ions (e.g., 25 mM KCl) and application of the test fraction buffer alone, i.e.,negative control, which as shown is Phosphate Buffered Saline PBS. Subsequently, responses to pharmacological agents, or test compounds can be recorded. Instantaneous changes to [Ca^2+^]_i_ can be monitored based on Fura-2 ratiometric emission intensity over time-course experiments comprising multiple stimuli (Figure 2).

Constellation pharmacology allows simultaneous recording of responses from thousands of diverse cells to various pharmacological agents. Subsequent analyses of these recordings provide the functional identification of each cell in a microscopic field [17,18]. Thus, constellation pharmacology combines the principles of fluorescence-based calcium imaging assays with a sequential application of different pharmacological agents and computational algorithms for the deconvolution of the cellular responses. The responses are cell-type specific and indicative of specific sets (constellations) of ion channels and membrane receptors expressed in each cell [19]. Thus, by the sequential application of different pharmacological agents, cell-specific “fingerprints” are recorded, which are then used to sort each cell into a cell class (Table 1). This methodology has been extensively used for the identification and characterization of both non-excitable and excitable cell populations from different loci of the nervous system [17,18,20,21,22]; for assessing neuronal functions in naïve animals [17,23]; for investigating the cell-specific changes in pathological conditions [24]; and for the screening and characterization of venom-derived peptides [25,26,27,28]. In this latter application, constellation pharmacology has a series of advantages compared to other screening methods, such as conventional calcium imaging. First, the simultaneous recording of ~2000 cells during the application of a given set of pharmacological agents (or tracers), followed by response analyses allows for the classification of each cell in the culture well [19]. This cell identification is then used to elucidate the cell type specificity for unknown test compounds, such as venom constituents, with regard to respective indirect effects, namely amplification (phenotype A), inhibition (phenotype B), and direct effects (phenotype C) on the intracellular calcium level detecting Fura-2 signal elicited by a repetitive stimulus (e.g., 25 mM KCl pulses). A limitation of this calcium signaling reporter method is that, in some cases, additional work may be required to explicitly identify the target receptor(s). Thus, elucidation of the specific type of receptors driving elicited phenotypes may require additional work using specific and directed protocols with specific pharmacological agents. Further, the identification of more complex pathways, such as those involving G-protein coupled receptors (GPCRs), can involve single-cell analyses. However, since the cells are maintained alive during the constellation pharmacology experiment, they can be further subjected to other assays that could help elucidate specific targets and mechanisms [21].

### Phenotypic Characterization of Test Compounds

**Figure 3:**
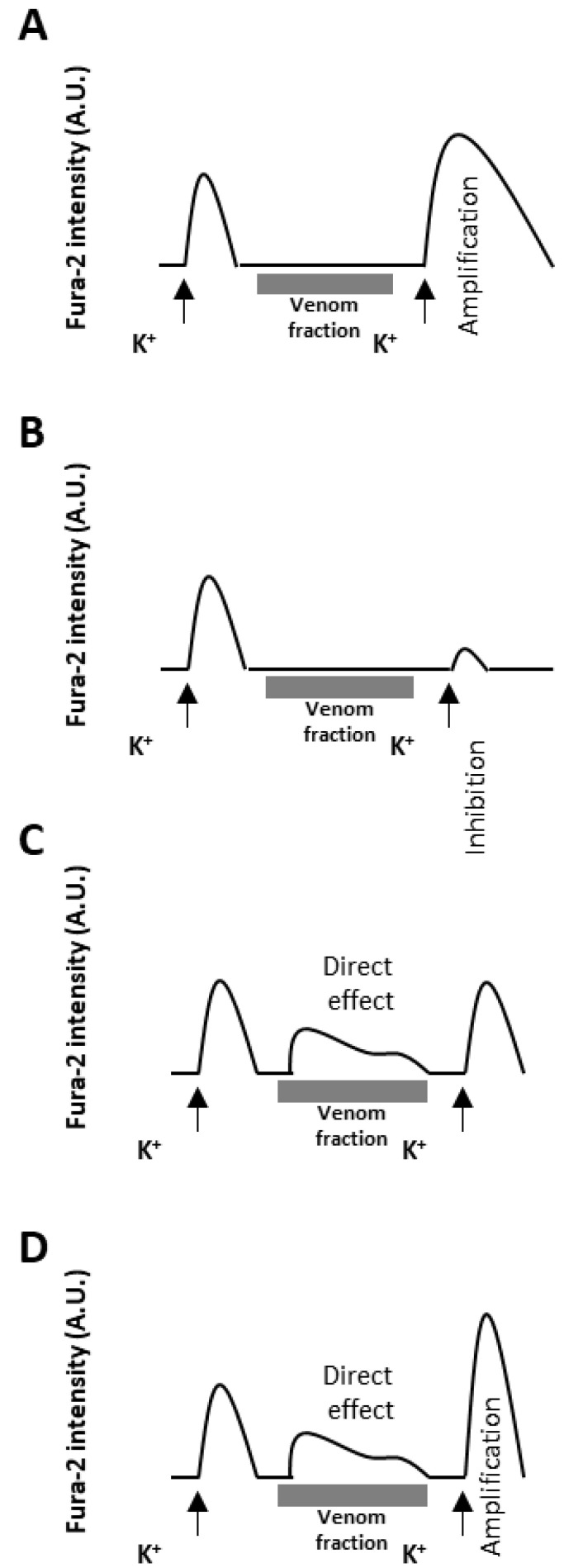
Representation of the different phenotypes evoked by venom fractions. *X*-axis is time in minutes and *Y*-axis represents the ratiometric emission of the Fura-2 excited at 340 nm and 380 nm. (**A**) Phenotype A: amplification of the KCl-induced Fura-2 signal. This phenotype could be elicited by voltage-gated potassium channel (VGKC) inhibitors, blockers of the voltage-gated sodium channel (VGSC) inhibition, or modulation of ligand-gated receptors; (**B**) phenotype B: inhibition of the KCl-induced Fura-2 signal. VGSC inhibitors or modulators of ligand-gated receptors are examples that could lead to this phenotype; (**C**) phenotype C: direct effect consisting of a spontaneous (independent from the depolarization stimulus) increase in [Ca^2+^]_i_ upon incubation with the venom fraction. VGSC activators or ligand-gated receptor modulators could directly induce an increase in the influx of Ca^2+^; (**D**) example of dual effects. Complex venom fractions or isolated compounds could elicit a combination of phenotypes (typically A and C, or B and C) on the same cell. Some VGKC blockers, such as the conotoxin κM-RIIIJ, for instance, could cause both a direct effect and amplification [21].

The test venom fraction is applied to the cells and incubated in between two successive pulses of the depolarizing stimulus (e.g., 25 mM KCl) and the intensity of the calcium signal before and after the application is compared (Figure 3). If the signal following the incubation with the venom fraction is higher than the previous one, it is called an amplification (phenotype A); if the signal is lower, it is then considered an inhibition (phenotype B). Finally, direct effects (phenotype C) cause spontaneous increases in the Fura-2 signal reporting intracellular calcium concentration upon contact with the venom fraction [17,18,21,27,32]. These phenotypes can appear isolated or together in the same cell. For instance, complex mixtures could have constituents interacting with different targets and elicit different phenotypes on the same cell. Isolated peptides could also have dual effects on the same cells, such as the case of peptides blocking voltage-gated potassium channels (VGKC) that can cause both direct effects (phenotype C) and amplification of the KCl-induced depolarization (phenotype A) [21]. Given that most medusozoan venoms characterized to date contain potent pore-forming proteins, or “porins” [1,3,5,33], it is important to note that porins, calcium ionophores, and components that destabilize the cellular membrane lead to the disruption of the plasma membrane integrity, which is typically irreversible and leads to cell death and Fura-2 leakage [13].

Taken together, analyzing the cell-specific phenotypes of a venom-fraction elicited response allows preliminary elucidation of membrane receptor-specific targets and physiological mechanisms. Additionally, the live cells comprising the well in the constellation pharmacology assay can be further analyzed via assays, including single-cell transcriptomics, whole-cell electrophysiology, immunohistochemistry, etc. [21,32]. Thus, constellation pharmacology can be paired with functional transcriptomics as well as classical electrophysiological characterization, allowing for detailed screening of venom bioactive compounds with regard to molecular target identification and physiological effects [19,21].

While most extant venoms are recovered in fairly large quantities from venom glands and comprise a liquid (e.g., snake and cone snail venoms), cnidarians are defined by the presence of microscopic eversible organelles, termed cnidae. Upon discharge, the everting hollow tubule of a penetrant cnidae or “nematocyst” pierces tissue to impale prey and inject cnidae content or venom. During a sting event, each rigid collagen-walled nematocyst expels only picoliters of venom, which studies have shown to be a highly complex mixture comprising a lipid-rich semi-solid gel [4]. If the nematocyst capsules are simply pulverized using various methods, including glass bead mill agitation, the authentic venom content is highly contaminated with non-venom capsule-wall collagen proteins. Further, these medusozoan venoms have been shown to be incredibly labile and heat-sensitive, requiring rapid preparation at cold temperatures to retain full bioactivity [1,33]. Given these facts, optimal collagen-free cnidae venom recovery with full bioactivity, as well as rapid constituent fractionation without extensive dilution create impediments in conventional purification strategies. Previous studies were conducted to optimize the recovery of bioactive venom components utilizing freshly obtained live tentacles [1]; these uniquely optimized methodologies were utilized in this present study.

Worldwide sting incidence, morbidity, and mortality data show that cubozoan and *Physalia* species account for the majority of stings requiring medical intervention [34,35,36,37,38,39]. Despite the health burden associated with the reported millions of jellyfish sting events worldwide per year, controversy surrounds medical management. Sting management is hindered by a lack of thorough understanding of the pathophysiological mechanisms of medusozoan envenomations. While life-threatening envenomations have been correlated with indiscriminate cytolytic activity of rapid-acting pore-forming proteins, e.g., CfTX, CaTX, [1,2,3,6,7,8], and life-saving rapid-acting therapeutics have been developed [2,40], the presence of a broad array of lower molecular weight proteins, peptides and small molecular weight components have long suggested auxiliary mechanisms at work. Recently, constellation pharmacology has been used to accelerate the elucidation of physiological effects of multiple cone snail venom toxins [26,27,41]. In this study, we utilized comparable research strategies to interrogate medusozoan cnidae venoms to better understand their constituent bioactivities and to potentially inform new therapeutic approaches for short- and long-term sequelae from medusozoan envenomation.

## 2. Results

### 2.1. Identification of Pore-Forming Activity from Crude Venom Using Calcium Imaging

The most potent (albeit labile) and well-characterized components of cubozoan jellyfish venoms are the porins [1,6,7,8,33]. Cubozoan porins self-assemble in cellular membranes, creating pores (with inner diameters ranging from 12 nm for *Alatina alata* to ~25 nm for *Chironex fleckeri*) [1]. Perforation of cellular membranes leads to a dramatic initial influx of calcium along the concentration gradient, as the extracellular calcium concentration is much higher, leading to a spike in the Fura-2 signal prior to cell death. This activity is irreversible and precedes the loss of both the 340 nm and 380 nm excitation-based 510 nm emissions. Therefore, the pore-forming activity could interfere with the readout of calcium imaging-based assays, and it is imperative to distinguish such non-specific, leaky phenomenology from the targeted, specific effects on ion channels and receptors when screening medusozoan venom components [13].

**Figure 4:**
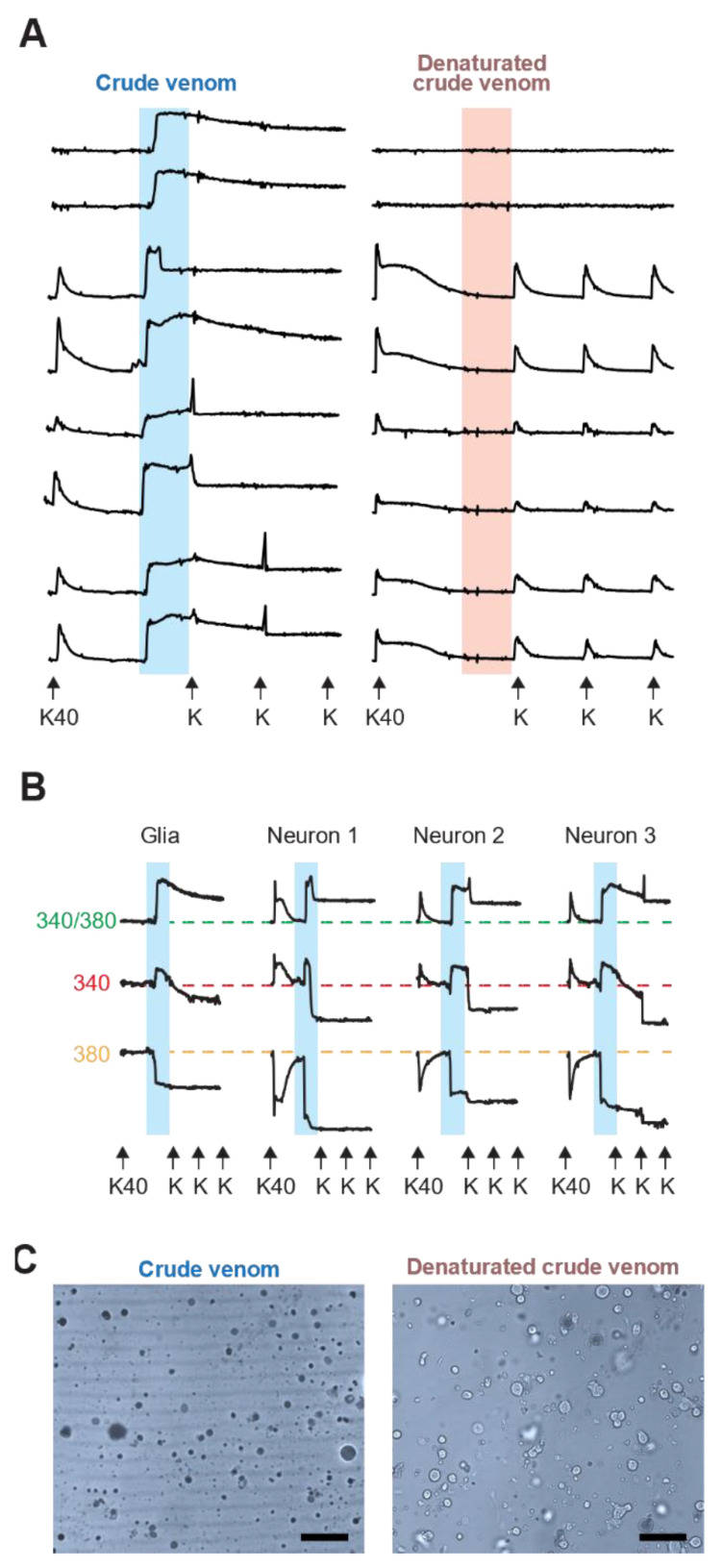
Porin activity of the *Alatina alata* crude venom using constellation pharmacology: (**A**) Representative traces of DRG cells exposed to crude venom for 3.5 min (blue and red boxes). The *X*-axis represents time. The *Y*-axis represents the ratiometric measurement of Fura-2 at 340 nm and 380 nm. K40 = 10 s pulse with 40 mM KCl; K = 10 s pulse of 25 mM KCl. Untreated crude venom (left) causes an increase in [Ca^2+^]i and sustained high ratiometric values. Crude venom denatured by heating at 100 °C for 10 min showed no effect; (**B**) comparison of the ratiometric signal (top row) with the deconvoluted signal of Fura-2 excited at 340 nm (middle row) and 380 nm (lower row); (**C**) bright field images of the DRG cells following 3.5 min treatment with crude or heat-denatured venom before and after the exposure with the crude venom. Scale bar = 100 μm.

To distinguish cubozoan pore-formation effects on the dorsal root ganglion (DRG) cell membranes, pulses of KCl were followed by application of active crude venom or fully heat-inactivated (100 °C for 10 min) crude *Alatina alata (A. alata*) venom (hemolytic unit (HU) [1] HU_50_ = 15.8 ng, crude venom protein concentration = 10 mg/mL) (Figure 4A). Specifically, the application of crude venom led to an immediate increase in the Fura-2 signal in the vast majority of the heterologous cells comprising the primary DRG culture. After an initial apex was reached, the ratiometric signal remained above baseline values throughout the duration of the experiment, even after several washes. In some neurons, the subsequent addition of extracellular KCl led to a further transient Fura-2 signal change for either one or two further KCl pulses. In contrast, fully heat-denatured crude venom showed no such effect on the DRG cells. Taken together, these results demonstrate the importance of cognizance of Fura-2 signal leakage, especially when examining cubozoan venoms.

In order to more precisely track the porin-related Fura-2 ratiometric signal, the 510 nm emissions based upon the individual excitation maxima of 340 nm and 380 nm were independently analyzed (Figure 4B). In a healthy intact DRG cell, when an extracellular KCl pulse was applied (“K40” 40 mM), neuronal cell membranes were depolarized, and the opening of voltage-gated calcium channels (VGCC) allowed the influx of Ca^2+^ ions. The increase in [Ca^2+^]_i_ led to an increase in the Fura-2 signal of calcium complexed Fura-2 at 340 nm and the concomitant decrease in the calcium-free Fura-2 signal at 380 nm, which resulted in an increase in emission of 510 nM based upon the ratio of excitation at 340 nm/380 nm. This was fully reversible after the KCl was washed out, with a return to baseline. The non-neuronal cells (glial cells) do not respond to the KCl pulse due to their lack of VGSC and VGCC [19,22,30]. In contrast to the KCl effect, while the addition of *A. alata* crude venom led to an immediate but irreversible rise in the [Ca^2+^]_i_ in both glial cells and neurons, the removal of the crude venom from the well, as well as subsequent KCl pulses, did not result in a return to baseline values. In the instance of one or two additional responses to subsequent KCl pulses after venom exposure, there may be incomplete permeabilization prior to cell death, and thus, membrane depolarization can still occur. However, past this point, the sustained elevation of the Fura-2 340/380 ratiometric signal no longer represents a reliable measurement of the cytosolic [Ca^2+^] [13]. Individual assessment of both calcium-bound and unbound Fura-2 shows both are leaking from the perforated cells with loss of both the 340 nm and 380 nm signals over time (Figure 4B). The fluorescence quantum yield and molar extinction coefficients for the calcium-bound Fura-2 (340 nm) are higher [42], and the calcium-bound molecule disperses more slowly than calcium-free Fura-2 (380 nm) after cell death, causing persistently higher 340/380 ratios as the overall intensity decreases. Thus, with irreversible responses, it is imperative to examine the individual Fura-2 signals.

Morphologically, exposure to crude-venom led to cell swelling; no changes in cell dimensions were observed after exposure to the fully heat-inactivated venom. Pore-forming toxins allow water to enter the cell along the osmotic gradient, leading to a rapid increase in cell volume, i.e., “swelling”. Finally, a conventional Trypan Blue exclusion test of cell viability was performed; <0.1% of cells remained viable following crude venom incubation as compared to >99% of cells exposed to fully heat-inactivated venom (Figure 4C).

These results highlight the importance of effective identification, separation and removal of cell membrane-disrupting components from other cnidarian venom components in order to clearly determine the specific molecular targets of these other constituents.

### 2.2. Assessing the Activity of Selected Venom Component Fractions

After analyzing the confounding effects of membrane disruptors and porins, i.e., the pore-forming activity phenotype, in the calcium imaging assay, we recorded the activity of venom fractions lacking the porins to identify specific activities on DRG cells.

**Figure 5:**
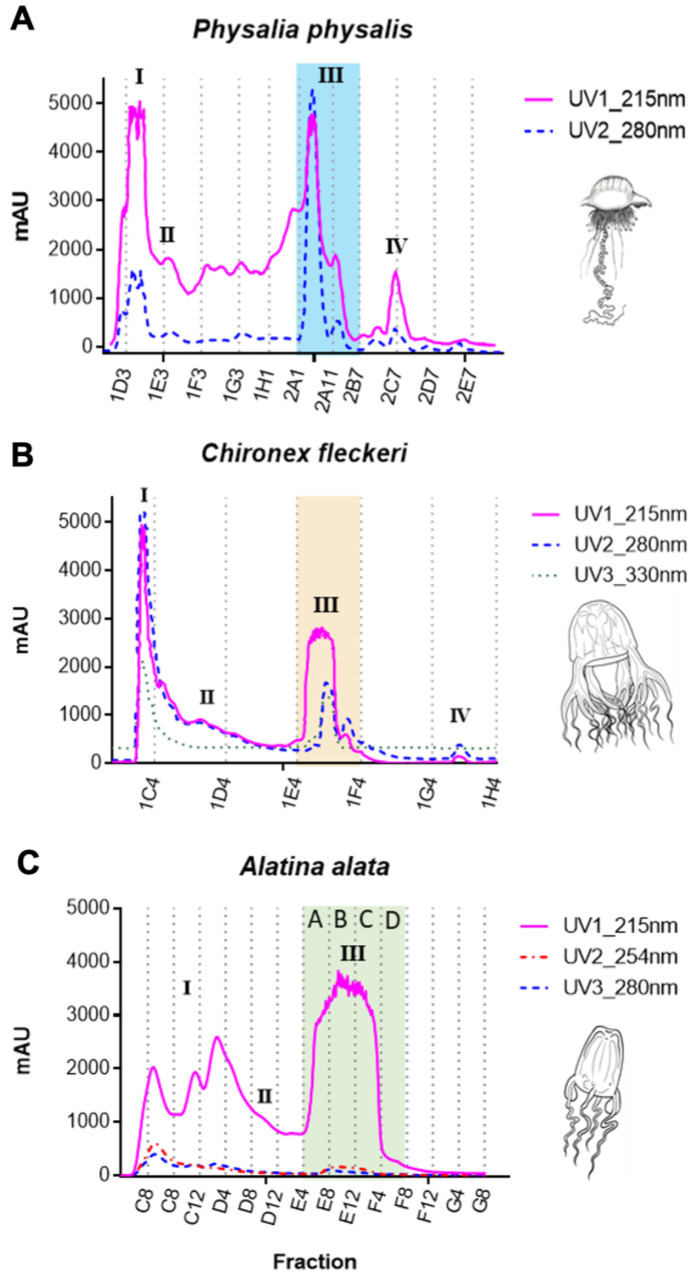
Size fractionation chromatograms of cnidarian venom preparations using size exclusion column high-performance liquid chromatography (SEC-HPLC). Major peaks are labeled: Peak I, molecular weight (MW) of ~250 kDa to 60 kDa comprising large proteins; Peak II MW ~50 kDa to 30 kDa comprising proteins including porins; Peak III MW ~15 kDa to 5 kDa comprising peptides and molecules investigated in this study; Peak IV MW ~3 kDa to 1 kDa. (**A**) *P. physalis* venom; (**B**) *C. fleckeri* venom; (**C**) *A. alata* venom. The fractions highlighted in Peak III were analyzed as subfractions (Peak III A–D) as well as pooled together.

As described in the methods, tentacle nematocysts from freshly caught *Physalia physalis (P. physalis)*, *Chironex fleckeri (C. fleckeri)*, and *A. alata* were recovered and processed to yield capsule-wall collagen-free crude venom, which was subsequently size-fractionated into 96-well plates using analytical high-performance liquid chromatography (HPLC). Previous work [1] described quantitation methods also used in this study to assess the resultant venom bioactivity. Peak III pooled or individual venom fractions (size exclusion: HPLC fractions < ~20 kDa MW, Figure 5) from *P. physalis*, *C. fleckeri*, and *A. alata* were applied to mouse DRG cells to assess intracellular calcium responses using pulse-chase protocols comprising constellation pharmacology (as described in Section 1 and Section 5). In general, the experimental protocol comprised the addition of the described concentration ranges of pooled, size-fractionated 96-well plate venom fractions from the respective jellyfish species between exposure to successive extracellular KCl pulses in the well chamber with buffer washes in between. Additionally, different agonists were applied to individually characterize the temporally tracked heterologous cells comprising the DRG primary cell culture preparation. Briefly, specific agonists target select types of transient receptor potential (TRP) channels: allyl isothiocyanate (AITC) is an agonist of TRPA1 receptors; menthol is an agonist of the TRPM8 receptor; and capsaicin is an agonist of the TRPV1 receptor [43,44,45]. Additionally, incubation with the conotoxin κM-RIIIJ from *Conus radiatus* was also applied to identify specific cell classes, as previously reported [17,21,46].

**Figure 6:**
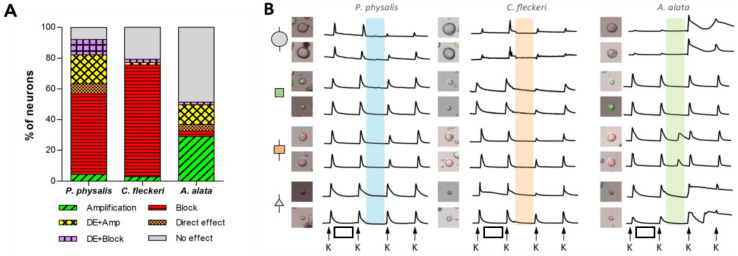
Overall comparison of the effect of venom Peak III from three medusozoan species using constellation pharmacology: (**A**) proportion of different neuron responses; (**B**) profile of representative cells comprising panel A. Two profiles of each neuron class are shown. From top to bottom: large-diameter unlabeled neurons (large gray circle), medium-diameter GFP-positive neurons (peptidergic nociceptors) (green square), medium-diameter IB4-positive neurons (non-peptidergic nociceptors) (orange square), and small-unlabeled neurons (gray triangle). The *x* axis represents time. The *y* axis represents the ratiometric measurement of Fura-2 at 340 nm and 380 nm. K = 10-s pulse with 25 mM KCl. Vehicle control: white box = 3.5 min incubation with PBS. The incubation with the venom fractions is represented by the blue (*P. physalis*), orange (*C. fleckeri*), and green (*A. alata*) boxes.

As described in Figure 1 and Figure 3, responses to venom fractions were scored as: “phenotype A”, indirect amplification (if the venom fraction increased the subsequent cell response to KCl pulses); “phenotype B”, indirect block or inhibition (if the venom fraction reduced or abolished the cell response to subsequent KCl pulses); “phenotype C”, direct effect (if the fraction led to a transient increase in intracellular calcium); or no effect. At higher concentrations, Peak III exposure resulted in robust and diverse responses in 92.1% (*P. physalis*), 79.3% (*C. fleckeri*), and 51.4% (*A. alata*) of neurons (Figure 6A). More specifically, while exposure to Peak III fractions from *P. physalis* and *C. fleckeri* venoms led to indirect inhibitory effects (phenotype B), Peak III *A. alata* fraction exposure resulted in phenotype A.

In all three species, a subset of neurons showed a dual response involving the direct effect (phenotype C) together with either amplification (phenotype A) or inhibition (phenotype B). These accounted for 28.8%, 3.7%, and 14.5% of the total neurons for *P. physalis*, *C. fleckeri*, and *A. alata*, respectively. Figure 6B summarizes representative calcium-imaging responses of four different cell classes to the specific venom fractions. The effects observed on DRG cells were reversible and transient, persisting for one, or at most two, KCl pulses after venom fraction application. Finally, the venom fractions from all species demonstrated concentration-dependent responses, in which lower concentrations elicited responses in fewer cells but with more cell class specificity (Supplementary Tables S1–S3). These cell-specific, reversible effects following exposure to specific Peak III fractions thus stand in stark contrast to the nonspecific and irreversible porin activity. These results also demonstrate robust and diverse effects of venom fractions from three different medusozoan species, suggesting the potential for divergent evolution of their venom components.

### 2.3. Cell-Specific Effects Elicited by Peak III Fractions

**Figure 7:**
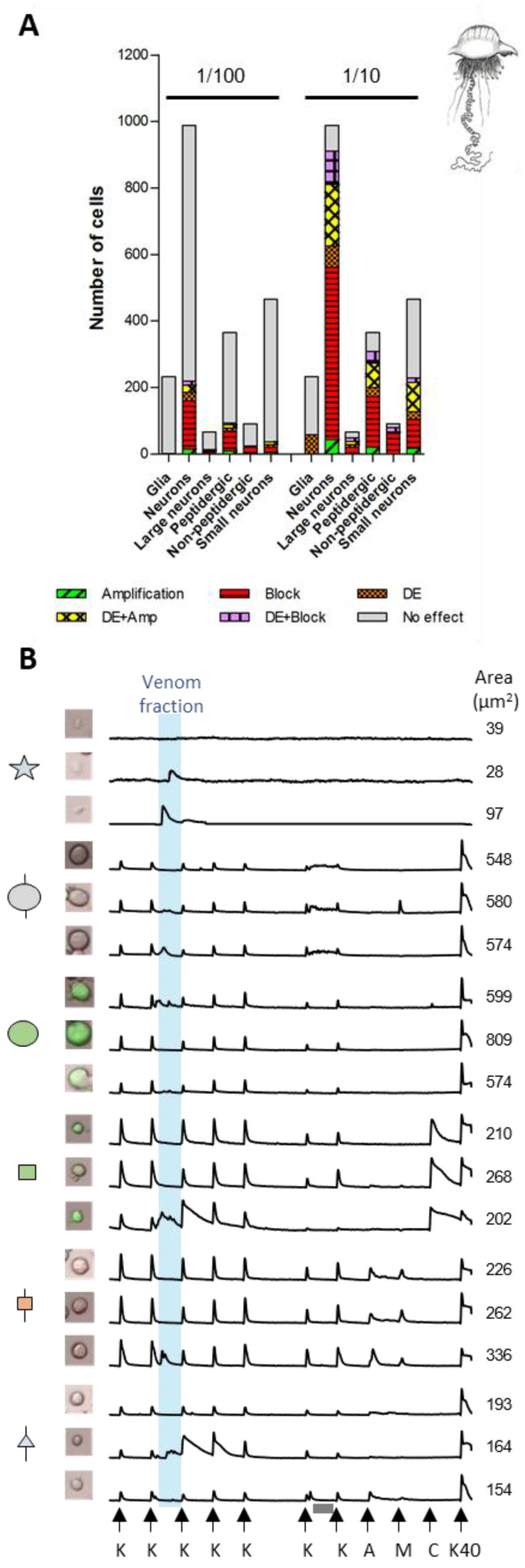
Effect of the Peak III pooled venom fractions from *Physalia physalis* on DRG cells: (**A**) proportion of different cell types responding to the peptide fraction of *P. physalis* using two different concentrations; (**B**) representative calcium traces from panel A exposed to the venom fraction for 3.5 min (blue box). Three profiles of each neuron class are shown. From top to bottom: glial cells, large-unlabeled neurons (L1–L4), large-GFP positive neurons (L5–L6), medium-GFP positive neurons (peptidergic nociceptors), medium-size IB4 positive neurons (non-peptidergic nociceptors), and small-unlabeled neurons. The *X* axis represents time. The *Y* axis represents the ratiometric measurement of Fura-2 at 340 nm and 380 nm. K = 10 s pulse with 25 mM KCl; A = 10 s pulse of AITC; M = 10 s pulse of menthol; C = 10 s pulse of capsaicin; K40 = 10 sec pulse of 40 mM KCl. Vehicle control: white box = 2.5 min incubation with PBS. Grey box = 3.5 min incubation with the conotoxin κM-RIIIJ.

**Figure 8:**
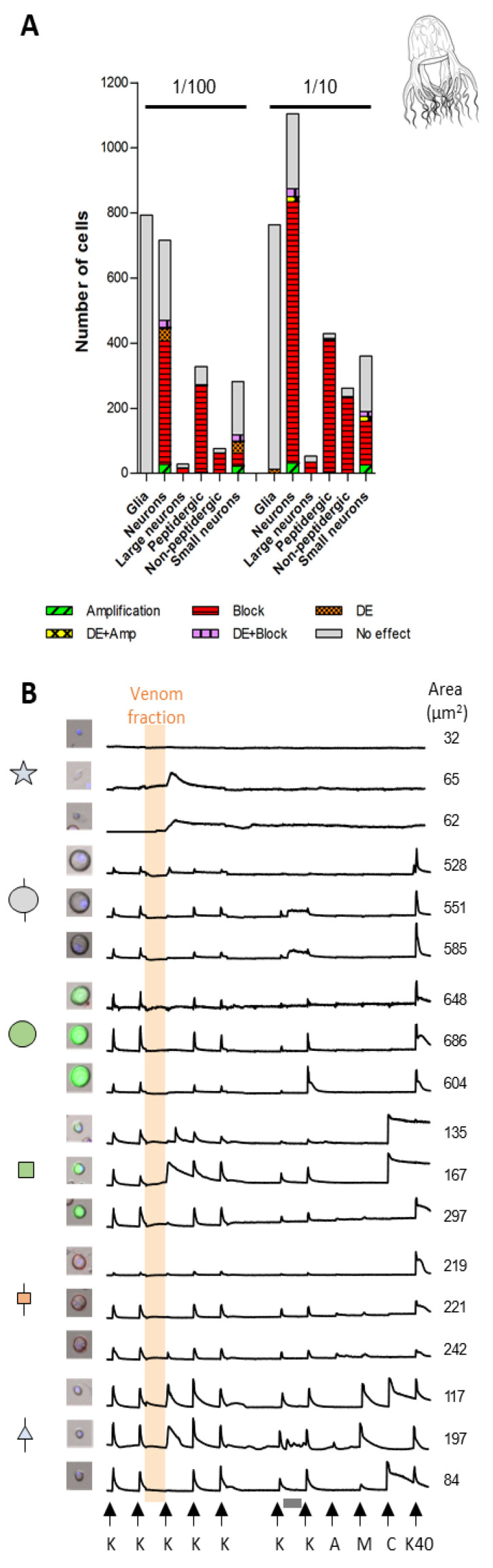
Effect of the Peak III pooled venom fraction from *Chironex fleckeri* on DRG cells: (**A**) proportion of different cell classes responding to the peptide fraction of *C. fleckeri* using two different concentrations; (**B**) profile of representative cells from panel A exposed to the venom fraction for 3.5 min (orange box). Three profiles of each neuron class are shown. From top to bottom: glial cells, large-unlabeled neurons (L1–L4), large-GFP positive neurons (L5–L6), medium-GFP positive neurons (peptidergic nociceptors), medium-size IB4 positive neurons (non-peptidergic nociceptors), and small-unlabeled neurons. The *X* axis represents time. The *Y* axis represents the ratiometric measurement of Fura-2 at 340 nm and 380 nm. K = 10 s pulse with 25 mM KCl; A = 10 s pulse of AITC; M = 10 s pulse of menthol; C = 10 s pulse of capsaicin; K40 = 10 s pulse of 40 mM KCl. Vehicle control:white box = 3.5 min incubation with PBS. Grey box = 3.5 min incubation with the conotoxin κM-RIIIJ.

**Figure 9:**
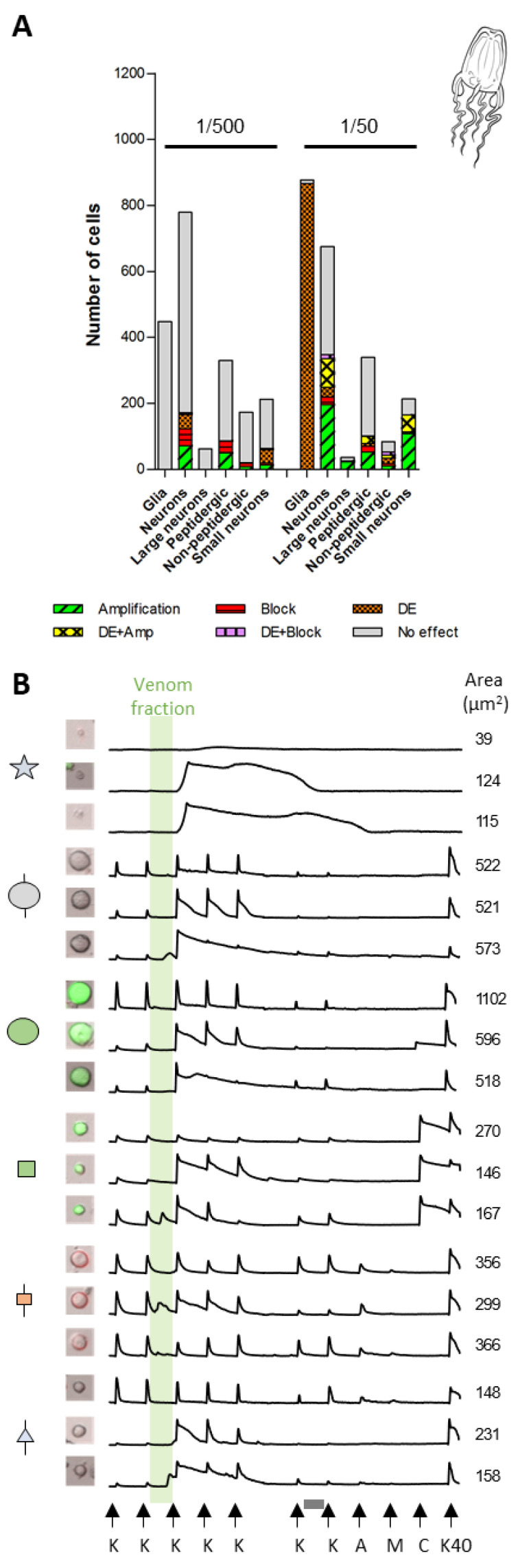
Effects of Peak III pooled venom fractions from *Alatina alata* on DRG cells: (**A**) proportions of responses in different cell classes at two different concentrations; (**B**) time-course Fura-2 signal intensity response profile of representative cells from panel A exposed to Peak III for 3.5 min (green box). Three profiles of each neuron class are shown. From top to bottom: glial cells, large-unlabeled neurons (L1–L4), large-GFP positive neurons (L5–L6), medium-GFP positive neurons (peptidergic nociceptors), medium-size IB4 positive neurons (non-peptidergic nociceptors), and small-unlabeled neurons. The *X* axis represents time. The *Y* axis represents the ratiometric measurement of Fura-2 at 340 nm and 380 nm. K = 10 s pulse with 25 mM KCl; A = 10 s pulse of AITC; M = 10 s pulse of menthol; C = 10 s pulse of capsaicin; K40 = 10 s pulse of 40 mM KCl. Vehicle control: white box = 3.5 min incubation with PBS. Grey box = 3.5 min incubation with the conotoxin κM-RIIIJ.

The venom fractions of the three jellyfish species elicited diverse and cell class-specific phenotypic responses (Figure 7, Figure 8 and Figure 9 and Supplementary Tables S1–S3). One of the most striking differences was observed in the DRG cell population unresponsive to KCl pulses (non-excitable glial cells). At lower concentrations, Peak III fractions of the different species showed no significant effect on glial cells, while higher concentrations elicited a direct increase in [Ca^2+^]_i_ of varying amplitude and kinetics. The 1/10th dilution of Peak III from *P. physalis* affected ~25% of the glial cells (Figure 7A). A rise of the Fura-2 signal occurred within the incubation time and generally did not persist past the application of the subsequent KCl pulse (Figure 7B). Peak III from *C. fleckeri* affected very few glial cells (~2%) (Figure 8A). In this case, the rise of the Fura-2 signal occurred right after the venom fraction was washed out and lasted for approximately 5 min (Figure 8B). In contrast, most of the *A. alata* Peak III exposed glial cells (>95%) exhibited increased [Ca^2+^]_i_ beginning after the venom fraction was washed out and persisted for up to ~40 min (Figure 9A,B).

Differential activity with regard to DRG neuronal cell class effects was also observed. As mentioned above, venom fractions from *P. physalis* and *C. fleckeri* predominantly elicited inhibitory effects (Figure 7A and Figure 8A). At higher concentrations, these effects were ubiquitous (Supplementary Tables S1 and S2). At lower concentrations, the venom fraction from *P. physalis* predominantly affected the medium-diameter neurons, both GFP-positive (peptidergic) and IB4-positive (non-peptidergic nociceptors). In particular, the subpopulation of TRPV1-positive peptidergic and TRPA1-positive/TRPV1-negative non-peptidergic nociceptors were targeted. In contrast, the lower concentration of the venom fraction from *C. fleckeri* evoked responses in most cell classes, aside from moderate effects on small-diameter, TRPM8-positive neurons (since TRPM8 receptors are also activated by cold stimuli, these cells were identified as cold thermosensors [17]), and showed little to no effect on other small-diameter neurons (expressing TRPA1 and TRPV1) Interestingly, in addition to inhibitory effects, the lower concentration of Peak III fractions from *C. fleckeri* had direct effects on cold thermosensors. Further, at the higher concentration, a significant amount of this cell class showed amplification (alone or in combination with direct effects) (Supplementary Table S2).

Exposure to Peak III pooled venom fractions from *A. alata*, in contrast, predominantly elicited amplification of the KCl-induced influx of [Ca^2+^]_i_ (Figure 9 and Supplementary Table S3). At the higher concentration, this effect was seen in most of the cell types. Phenotype C occurred in medium-diameter TRPA1^+^ and TRPV1^−^, non-peptidergic neurons, and moderate dual effects (direct effect plus amplification) were observed in most of the non-peptidergic and small-diameter neurons. At the lower concentration, phenotype A remained in all cell types, with the exception of large-diameter neurons. Phenotype C was concentrated in the cold thermosensors at the lower concentration. Interestingly, phenotype B was observed on medium-diameter neurons (Supplementary Table S3).

These results indicate that the venom size-exclusion HPLC fractions eluting below approximately 20 kDa (Peak III) have species-specific bioactive components with diverse activity on DRG cells. The distinct cell populations and their unique responses elicited by the venom fractions indicate a diversity of targets (i.e., ion channels and membrane receptors) for the bioactive components.

### 2.4. Constellation Pharmacology as Bioactivity-Driven Isolation Platform for Jellyfish Venom

**Figure 10:**
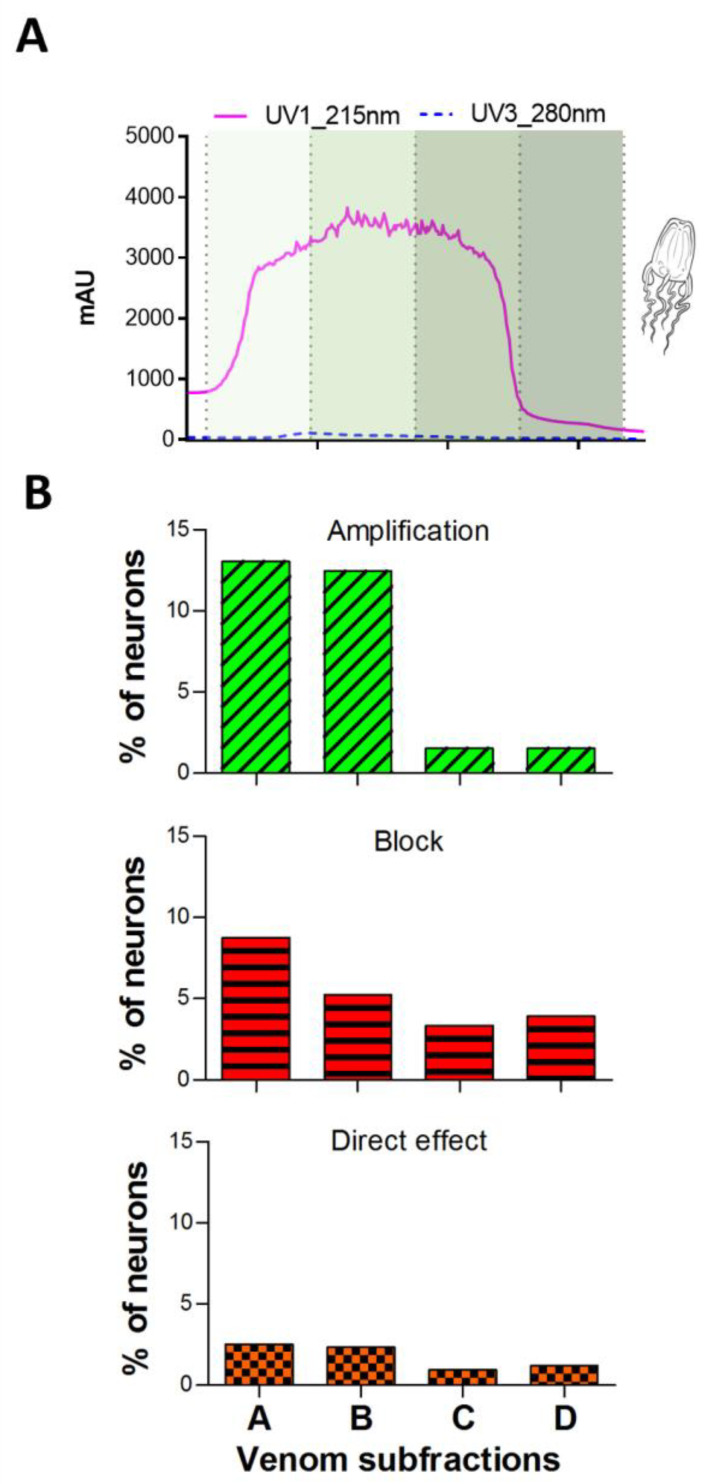
Bioactivity-resolution within Peak III of *A. alata* using constellation pharmacology: (**A**) chromatogram of individual and pooled fractions comprising Peak III of *Alatina alata* venom shown in Figure 4. Each shade of green (A–D) represents the different subfractions used for the constellation pharmacology assay; (**B**) proportion of neurons responding to the different subfractions classified by type of response.

The constellation pharmacology platform was used to further analyze four pooled subfractions (A–D) comprising Peak III from *A. alata* venom (Figure 10A). Figure 10B shows a quantification of the different DRG cell responses to the subfractions. The different phenotypes are individually presented to follow specific activities. However, dual responses were also elicited by Peak III subfractions (see Supplementary Table S4). The application of both subfractions A or B elicited amplification of around 12–13% of the cells, whereas subfractions C and D showed amplification on less than 3% of the cells. The inhibitory effect ranged between ~4 and 8%, with no significant difference between the subfractions. Similarly, direct effects were homogenous in their distribution across the subfractions, with the effects occurring in less than 3% of the neurons. This suggests that the venom component(s) that causes the characteristic phenotype A response of the *A. alata* venom fraction (amplification of the KCl-driven signal) is principally found in subfractions A and B (the first half of Peak III).

## 3. Discussion

The present work demonstrates marked advances in elucidating medusozoan venom component bioactivity, made possible by the combination of two technical innovations—(A) a live-tentacle cnidae isolation and rupture protocol to maximize the recovery and activity of the full suite of bioactive components of jellyfish venoms, and (B) the use of constellation pharmacology for monitoring bioactivity as detailed in the following sections. For the latter to be effective, the potent pore-forming venom component must be separated from other bioactivities.

### 3.1. Medusozoan Venom Recovery

While whole anemone bioactivity-based purification has led to the discovery of multiple families of cell membrane channel-specific ligands [47,48,49,50] and other bioactive compounds over the past 50 years [47,51], the teleological question of cnidae venom peptides and lower molecular weight constituents in medusozoans remains largely unexplored. A principle impediment to progress in the latter is the technical complexity inherent in the recovery of authentic “venom”, i.e., the complete suite of cnidae encapsulated constituents delivered into prey during envenomation (free from contaminants such as cnidae capsule wall collagen proteins, tentacular tissue, and microscopic marine biota). The venom injected into the tissue of prey or victim during a sting is the result of thousands to millions of separate transdermal impalement and venom expulsion events by penetrant cnidae or nematocysts. Each event involves the release of sub-nanoliter volumes and the physical trauma comprising “bullet-force” perforation [52] by the chitinous spine-laden discharging hollow tubule (cf. hypodermic-delivery device). In addition to the minute volume of venom in each structurally complex cnidae, the principle bioactivities, while immensely potent (active in ng to pg ranges), are highly heat labile. Thus, the recovery of sufficient quantities of the full suite of molecules and optimally active venom requires unique approaches. For the most part, the field has eschewed such considerations [53,54,55,56,57,58,59,60,61,62] and findings have been limited to the characterization of relatively stable saline soluble proteins including metalloproteinases and phospholipases, as well as porins, albeit with greatly diminished specific activities (ug/mL vs. ng/mL) [53,59,61].

More specifically, various approaches aimed at recovering venom from tentacles have been published [1,53,54,55,56,57,58,63]. Most recovery methods use PBS, distilled water, or seawater to wash tentacles, followed by homogenization of the tentacles or centrifugation of recovered cnidae after incubation for hours to days in cold distilled water or seawater solution. The latter is termed “autolysis”, a process in which the tentacle begins to decompose and the mesoglea and tentacular muscle tissues dissociate. A certain number of cnidae spontaneously discharge during this “autolysis” step. Recovered cnidae are sometimes lyophilized or stored as frozen pellets, then ruptured using techniques such as grinding with siliconized glass mortar and pestle, glass-bead mill shaking, sonication, or exposure to freeze–thaw cycles with liquid nitrogen. The approaches using glass tissue homogenization [54] and sonication [53,55,56] result in less recovery of high molecular weight proteins and also contain tentacular cell debris, extracellular matrix components, and collagenous structural proteins of cnidae [1].

In contrast, the cnidae venom recovery process involving gentle rotation in 4 °C 1 M trisodium citrate and the use of a French Press did not contain notable quantities of structural or extracellular matrix proteins. In comparison to incubating tentacles in seawater or distilled water, gently rotating the tentacles in a citrate solution followed by sieving led to over 95% recovery of intact cnidae from tentacles. After cnidae rupture using a French Press pressure cell, immediate centrifugation removed the ruptured collagenous cnidae capsules and tubule structures, allowing the specific recovery of the viscous cnidae content, comprising the full suite of venom constituents, not just the saline soluble components in saline extraction methods. Finally, snap-freezing the venom in liquid nitrogen and storing at −80 °C preserves its bioactivity. This method of venom purification demonstrates the highest yield of venom constituents and higher specific activity per animal and nematocyst as compared to extant saline venom extraction methods [1].

### 3.2. Medusozoan Venom Bioactivity

The functional properties of medusozoan nematocyst venom constituents have been examined by a few investigators [58]. Metalloproteinases, porins and other high molecular weight proteins have been described biochemically and using functional assays [1,7,33,55,59,60,64,65]. Despite these advances, small molecular weight and peptide component characterization is lacking. As discussed above, venom isolation and purification protocols utilizing whole-animal alcohol extraction [66] or chemical simulation and ACN/TFA conditioning [51] in sea anemones do not effectively recover the cnidae contents of medusozoans due to the presence of self-polymerizing proteins and aggregating structural collagens. Further, steps in this methodology preclude venom constituents from analysis; for example, heat treatment resulting in denatured proteins and subsequent removal, or lipid-separation by acetone or dialysis [9]. Additionally, biomolecules that are not part of the authentic venom but are present in the whole animal are included in the final extract. By utilizing the whole-crude venom isolated by the Yanagihara and Shohet [1] method for fractionation, we minimize the loss of bioactive components for analysis. SEC-HPLC is a useful technique to isolate unknown compounds, as it does not require organic solvents that can alternative protein structures, affecting their activity [58]. Further, it can provide resolution of complex compounds without knowledge of specific proteins’ solubility and/or charge [58]. Direct assessment of the bioactivity from SEC-HPLC fractions of French Press-prepared venom in physiological saline can, therefore, be performed in whole-blood or blood-component assays and in artemia, mouse, crayfish, and piglet models. These assays are useful in characterizing venom fraction activities but are limited by the low overall yield of purified products. Despite utilizing a highly efficient recovery method and fractionation approach, the final mass of the venom components isolated is sample delimited, especially in contrast to other venomous species, such as snakes. This is where the high-throughput, high-content method of constellation pharmacology provides a means for assessing the bioactivity of complex difficult-to-obtain venoms. This platform requires minute volumes of venom fractions to simultaneously analyze the bioactivity in thousands of individual cells.

One of the challenges of utilizing calcium imaging-based assays such as constellation pharmacology when analyzing cnidarian venoms is to identify elicited phenotypes that could obscure the interpretation of the results. In particular, the presence of highly active porins during the screening process could lead to misinterpretation of the data, as the pore-formation in the cellular membrane leads to a spontaneous increase in the Fura-2 signal and could be mistakenly scored as direct effects (phenotype C). Three major factors should be considered when venom fractions or constituents elicit direct effects: reversibility, specificity, and probe leakage. As we demonstrated in this work, pore-forming activities are characterized by being irreversible (signal never returns to baseline values), nonspecific (majority of the cells responded), and leaky (Fura-2 leaked over time, as revealed following 340 nm and 380 nm signals individually). In light of these results, the presence, or suspicion, of porins or other components that could interfere with calcium imaging and signaling mandates the application of appropriate controls and adjusted protocols to dissect the activity of the different constituents. In this work, we demonstrated that the SEC-HPLC venom fractionation effectively separated the membrane-disrupting porins from peptide and low molecular weight venom components, as Peak III showed no pore-forming phenomenology. This allowed for the analysis of novel and cell-specific activities of low molecular weight venom constituents, a field essentially unexplored to date.

Using the constellation pharmacology approach, the relative activation or inhibition of neuronal and glial responses can be evaluated for venom fractions, elucidating their cell specificity, the type of response elicited, as well as their potency. Robust activity in 92.1%, 79.3% and 51.4% of neurons was observed following the application of 1/10th dilutions of *P. physalis*, *C. fleckeri*, and 1/50th dilution of *A. alata* venom fractions, respectively (Figure 5). Diverse and cell-type specific activities were found. Amplification of the calcium signal (phenotype A) was most prominent in response to *A. alata* venom, whereas inhibitory activity (phenotype B) occurred in ~30% and ~50% of neurons in response to *P. physalis* and *C. fleckeri* venoms, respectively. Finally, direct effects (phenotype C) were more common in *A. alata* (~45%) and *P. physalis* (25%) venoms. The excitatory and inhibitory effects on the neurons suggest the presence of multiple components targeting various pathways (Figure 1) that lead to the downstream modulation of calcium signal. These results correlate with published transcriptomics data [6,67,68,69,70], demonstrating the highly divergent nature of medusozoan venom composition, including small molecular weight proteins/peptides and molecules. Further, the activities of the Peak III fractions from representatives of the divergent cubozoan class orders of Chirodropida and Carybdeida (i.e., *C. fleckeri* and *A. alata*) are strikingly different.

The specific phenotypic response elicited in the DRG cells depends on the nature of the tested compound and the molecular target. Although these venom fraction components have not yet been fully elucidated, previous reports provide examples of molecular mechanisms that could drive the different phenotypes. If the compounds activate pathways that favor membrane depolarization, e.g., block of VGKC or activation or prolongation of VGSC, they will typically elicit amplification of the KCl-induced signal (phenotype A) [19,26,46]. Conversely, compounds that activate pathways that prevent or diminish membrane depolarization, e.g., blocking VGCC and VGSC, will block the KCl-induced signal (phenotype B) [18]. Further, venom components that cause a flux of calcium into the cytoplasm, e.g., by opening RF-amide receptors or blocking some subtypes of VGKC, constitute an example of a direct effect (phenotype C) [20,27]. Finally, the venom components could elicit complex effects, comprising multiple phenotypes. For instance, by interacting with Kv1.1/Kv1.2-containing VGKC of different composition, the conotoxin κM-RIIIJ elicits phenotype A, preceded or not by direct effects (phenotype C) of various characteristics (i.e., smooth or jagged rise of [Ca^2+]^_i_) [17,21]. Therefore, the type of elicited responses and cell-specificity of the Peak III fraction from jellyfish venom allows us to speculate on the possible effects caused by their constituents. The different medusozoan venoms tested have shown activities compatible with different types of paralysis, pain sensation, and direct effects similar to that elicited by RF-amide class toxins (see below).

**Figure 11:**
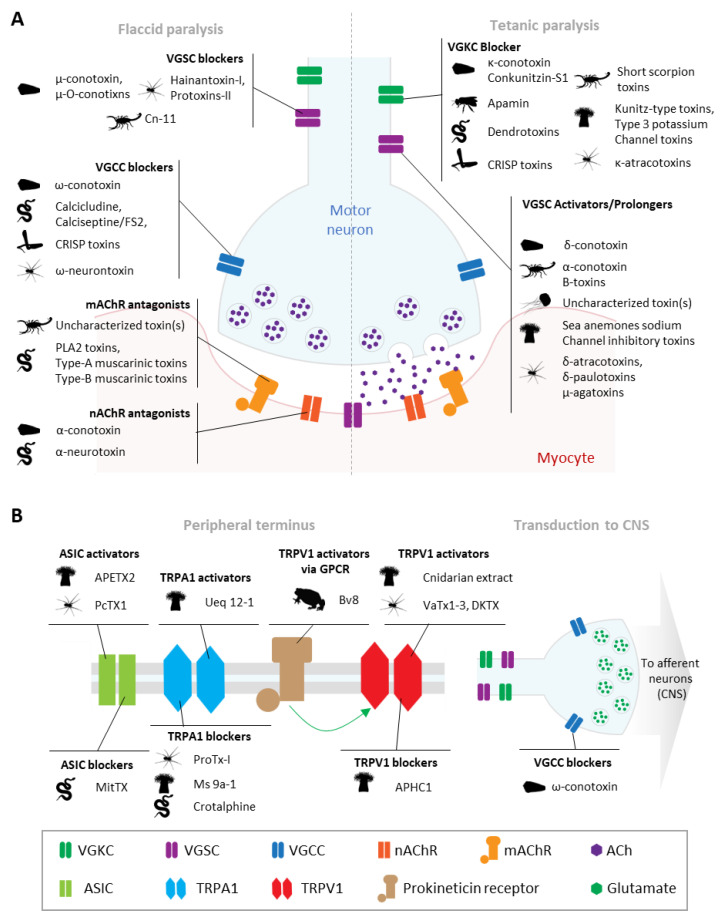
Venom-derived toxins in paralysis and pain: (**A**) Motor neuron–muscle synapse and toxin targets. Flaccid paralysis (left): the block of voltage-gated sodium channels (VGSC) inhibits the transmission of the electric signal to the neuronal terminus; the block of the voltage-gated calcium channels (VGCC) prevents the fusion of the synaptic vesicles for the release of the neurotransmitter acetylcholine (ACh). The block of the muscarinic (mAChR) and nicotinic (nAChR) acetylcholine receptors in the postsynaptic membrane inhibits the transduction of the signal into the myocyte. Tetanic paralysis: the block of voltage-gated potassium channels (VGKC) and the activation or activity prolongation of the VGSC result in more neurotransmitter release and overstimulation of the muscle. Modified from Trim and Trim 2013 [75]; (**B**) scheme of the pain-related receptors and the pain-signaling transmission to the central nervous system (CNS). Activation of the pain transducer receptors acid-sensing ion channel (ASIC) and transient receptor potential channel ankyrin 1 (TRPA1) and vanilloid 1 (TRPV1) lead to the activation of nociceptive neurons. The inhibition of either of these receptors has been related to analgesia. Additionally, the block of the VGCC at the nociceptor terminal abolishes the signal transduction to the CNS. Modified from Bohlen 2012 [76].

Many venomous animals impair their prey or predator by injecting paralytic toxins as part of their venom repertoire. The mechanism of paralysis can vary depending on the venom components’ excitatory or inhibitory effects on cellular membrane channel(s) or receptors(s) (Figure 11A), as well as the nervous circuitry that the affected neurons belong to [71], deriving in either flaccid or tetanic paralysis. For instance, μ-conotoxin (cone snails), BcIV, *Stichodactyla haddoni* toxin I-IV (SHTX-IV) and AmII (sea anemones) all target voltage-gated sodium channels (VGSC), resulting in flaccid paralysis [47,48,72]. Flaccid paralysis can also result from ⍵-conotoxins or α-conotoxins, which block VGCC and nAChR, respectively [72,73]. The blocking of VGCC, VGSC, or nAChR are possible pathways for the reduction in calcium signaling observed in constellation pharmacology (phenotype B). Alternatively, tetanic paralysis can occur as a result of blocking VGKC, as in the case with the *κ*-conotoxins from cone snails and BDS-I, and SHTXI-III from sea anemones [48,72,73,74] along with the activity on other ion channels and receptors. In constellation pharmacology, the venom fraction blocking VGKC would result in the amplification of the calcium signal (phenotype A).

Venoms can also target nociceptive pathways, causing pain in prey or predators (Figure 11B). The activation of TRPV1 or acid-sensing ion channels (ASICs) generates action potentials in pain-processing regions of the spinal cord and brain [76]. Certain venom components have been demonstrated to target another transient receptor potential channel, TRPA1, also implicated in pain sensation [77,78,79]. Conversely, some venom constituents affect analgesia by blocking these same membrane receptors [76]. Cnidarian venom extracts have been shown to affect TRPV1 receptors [76,80], causing envenomation-associated pain, but have not been previously characterized in terms of their effect on neuronal and non-neuronal DRG cells.

Phenotype C effects result in the direct amplification of calcium signaling following the administration of venom fractions. This phenomenon has been observed and described in a family of conopeptides belonging to the RF-amide class toxins [27,81]. RF-amides are characterized by a carboxy-terminal arginine and amidated phenylalanine residues, although the amidated amino acid can vary [81]. Both CNF-Vc1 and CNF-Ep1, isolated from *Conus victoriae* and *Conus episcopatus*, respectively, induced hyperactivity of larval zebrafish at low concentrations and paralysis at higher concentrations [27]. In concordance, CNF-Ep1 addition to mouse DRG demonstrates amplification of calcium signaling (phenotype A) at lower concentrations (1 μM) in select peptidergic nociceptive neurons and non-selective direct effects (phenotype C) in subsets of all cell types at increased concentrations (10 μM) [27].

As mentioned above, similar behaviors were observed for the tested jellyfish venoms fractions. The *P. physalis* venom fractions demonstrated effects from all three classes in all of the DRG neuronal cells (Figure 7), predominantly inhibiting (phenotype B) calcium signaling in medium-diameter neurons. Both TRPV1-positive peptidergic and TRPA1-positive nonpeptidergic nociceptors were notably affected, demonstrating potential analgesic properties. Additionally, transient direct effects were observed in a quarter of glial cells in response to the higher concentration of *P. physalis* Peak III.

*C. fleckeri* Peak III fractions also predominantly resulted in class B inhibition of calcium signaling (Figure 8) in neuronal cells but did not affect glia. The reduced signal could be due to blocking voltage-gated calcium or sodium channels; other effects such as GPCR-mediated signaling can also be implicated. The use of pharmacological calcium channel blockers did not prevent calcium influx [54] or acute cardiovascular collapse with administration of a saline extract type of lyophilized *C. fleckeri* cnidae [82,83]. It is more likely, therefore, that the sodium channels or GPCR receptors are implicated in the neuronal responses observed. However, activity on DRG-specific calcium channels cannot be ruled out. Although the dominant effect was inhibitory (class B), direct effects and signal amplification were also seen in cold thermosensor neurons at 1/100 and 1/10 concentrations of *C. fleckeri* Peak III fractions, respectively. Further analysis of individual HPLC fractions through constellation pharmacology will elucidate the specific venom components/peptides responsible for these diverse effects.

*A. alata* Peak III venom fractions interestingly exhibited concentration-dependent effects comparable to the conorfamides, with a higher proportion of direct and dual effects (amplification and direct effects) at the higher concentration (1/50 dilution), but less of these activities at the lower concentration (1/500 dilution). These effects were seen in peptidergic nociceptive neurons, as well as small-diameter neurons. Finally, persistent (40 min) effects were seen in glial cells following Peak III venom fraction addition despite washing. This intriguing glial cell effect also merits further investigation.

As described in Figure 10, the subfractions of *A. alata* venom from Peak III demonstrated diverse effects. The amplification effects were concentrated in subfractions A and B, indicating that a specific venom component/peptide caused this outcome. Transcriptomics (Yanagihara and Semmes, unpublished data) have shown the presence of Kunitz-type serine protease inhibitor C, which could theoretically act as a K-channel inhibitor, resulting in an amplification of the calcium signal. In contrast, the inhibitory and direct effects were homogenous and low across the four subfractions. Transcriptomic approaches [6] (Yanagihara and Semmes, unpublished data) demonstrate the presence of Kunitz-type serine protease inhibitor C2 (K-channel blocking, class A amplification) and RFamides (class C direct effects) in diverse medusozoan expressed venoms. Edman sequences (Yanagihara and Semmes, unpublished data) of N-termini of various peptides comprising Peak III in this study show homology to transcriptomic sequences. This encouraging correlation of “top down” (transcriptomic-based) data with “bottom up” bio-activity (biochemical purification and bioassay activity-based) provides a useful avenue forward. The next steps, thus, include comparisons of *A. alata* venom peptides to sequences of known channel-targeting peptides, followed by specific peptide synthesis and activity reassessment. Other channel-targeting peptides reported in anemones include the following. SHTXI-III demonstrated crab-paralyzing activity and were found to inhibit potassium channels [48]. The crab-lethal SHTX-IV was identified as a member of the type-2 sea anemone sodium channel toxin family [48]. Gigantoxin indirectly activated TRPV1 channels by the EGF receptor/PLA2 and downstream pathway [50]. Am II and BcIV share a similar structure to APETx2, a specific blocker of ASIC3 channels [47]. BcIV also shares structural components with the type 1 sodium channel toxins of sea anemones, as well as the acid-sensing ion channel effectors APETx1 [49].

The venom fractions from the three species tested affected subpopulations of nociceptor neurons by either blocking (*P. physalis* and *C. fleckeri*) or amplifying (*A. alata*) their activity. These results suggest the presence of pain modulators in the jellyfish venoms that could be useful for elucidating the mechanism of sting-induced pain as well as in natural product discovery of analgesic compounds. Ongoing research is focusing on isolating, sequencing, and further functionally characterizing individual bioactive jellyfish venom constituents and their molecular targets towards elucidating extant mechanisms of sting pathophysiology, as well as developing novel diagnostic and therapeutic tools [84]. For instance, here we demonstrated that pooled Peak III fractions from both *P. physalis* and *C. fleckeri* reduce the excitability of specific populations of peptidergic and non-peptidergic nociceptors, suggesting the presence of venom components that could potentially have therapeutic properties for treating pain. Conversely, the Peak III fractions from *A. alata* increase the excitability of nociceptors, indicating the contribution of small molecular weight components (previously unknown) that could help explain the pain sensation caused by their sting.

### 3.3. Future Directions

Future directions: (1) constellation pharmacology will be used to direct individual fraction constituents’ purification; (2) N-terminal Edman sequencing will be performed on bioactive peptides to screen extant transcriptomic (unpublished) libraries to pull out full sequence information; (3) synthetic peptides will be prepared based upon steps 1 and 2 and re-examined using constellation pharmacology; (4) additional studies will be performed to finely resolve cellular and molecular targets, including nAChR, TRP channel, etc., and ion channels, including K-channels, Na-channels, and Ca-channels; (5) known antagonists of channels (ex. TTX, block mammalian Nav channels [85]) will be utilized to validate the target specificity of venom fractions.

## 4. Conclusions

One enormous advantage of the constellation pharmacology platform is the ability to assess the multiple bioactivities present in a complex mixture without having to individually purify each component. Indeed, multiple mixtures can be assayed in a single one-hour experiment; in effect, both the ligands being assessed, as well as the molecular targets whose activities can be detected, are massively multiplexed. Thus, an overview of all of the potential bioactivities present in an individual venom is rapidly obtained, which allows efficient prioritization of which of the diverse bioactive venom components detected should be prioritized for a more thorough characterization. The present work demonstrates the immense utility of an optimized venom isolation and separation protocol, in combination with constellation pharmacology-based examination, to systematically screen the bioactivity of venom constituents from three important medusozoan species. This constellation pharmacology-based examination of three medically important medusozoan peptide- and small molecular weight- venom species demonstrates the enormous value of this approach for systematic screening of biomedically relevant activities. By analyzing small molecular weight components following separation from the pore-forming protein, it is possible to discern unique, concentration-dependent mouse DRG cellular responses, apart from cell death-induced Fura-2 signal. Notable cell-specific and diverse effects were found among three medusozoan Peak III (5–15 kDa) venom fractions. Further work is merited to continue to examine individual fractions, conduct peptide isolation and sequencing proteomic efforts together, and to integrate these data with top-down transcriptomic approaches (Yanagihara and Semmes, unpublished data) that have already revealed a suite of proteinaceous constituents, including families of RF- and GLW-amide constituents, and Kunitz domain-containing proteins.

## 5. Materials and Methods

### 5.1. Jellyfish Collection and Venom Preparation

**Figure 12:**
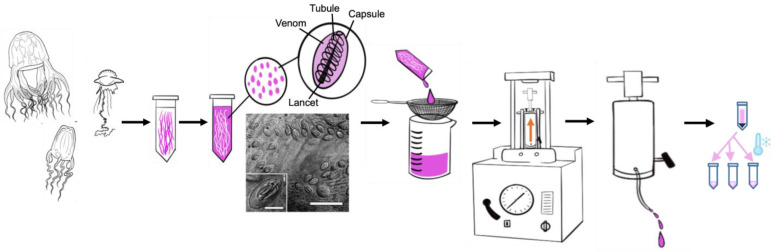
Jellyfish collection, intact cnidae recovery and venom preparation, as described in 5.1. From left to right (arrows denote steps in this process), schematic images of *Chironex fleckeri*, *Alatina alata* and *Physalia physalis*; schematic representation of freshly excised tentacles (pink) in chilled 1 M trisodium citrate; spontaneously shed intact cnidae are represented as pink ovals; light micrograph of *Alatina alata* eurytele [86] in live replete tentacle (scale bar 65 micron, inset 15 micron); 0.5 mm mesh sieving of citrate solution of tentacles to remove shed intact cnidae from depleted tentacles; pelleted cnidae are then ruptured in a chilled pressure cell disruptor (French Press); complete ruptured slurry is quickly centrifuged to pellet collagenous structural components (capsule walls and tubules), total cnidae content venom (supernatant) is then aliquoted and snap frozen in liquid nitrogen [1].

Adult *Chironex fleckeri* (~20 cm bell diameter) were collected along the Australian coast, and *Alatina alata* (~7 cm bell diameter) and *Physalia physalis* (~10 cm *float length*) were collected along the leeward and windward coasts of Oahu, respectively. As described previously [1], tentacles from freshly caught live animals were excised from each animal and placed into 4 °C 1 M trisodium citrate solution, in at least 7 volume excess (tentacle to 1 M citrate) [1] (Figure 12). Gentle rotation in cold 1 M trisodium citrate leads to slight tentacle mesoglea tissue contraction with the expulsion of fully intact undischarged cnidae. Live tentacle squashes were performed in seawater to allow for quantitation of cnidae packing density and diversity. During the course of cold citrate rotation, tentacle sections and citrate solution were examined microscopically to assess progress in cnidae recovery. This process was continued until over 95% of the cnidae had been recovered from the tentacles. The solution replete with undischarged cnidae was then sieved from the fully denuded tentacles. Yields were quantified for each preparation and typically resulted in an average of 1 × 10^6^ cnidae per adult *Alatina alata* (with approximately 2 mL tentacle displacement volume) [as described previously, 1] and 100 × 10^6^ cnidae per 20 mL displacement volume of fresh adult *Chironex* sp tentacles. Yields from *Physalia physalis* were approximately 1 × 10^6^ cnidae per 4 mL tentacle displacement volume. Consistent yields were observed over multiple years and locations when working with live freshly caught adult medusozoa. Yields dropped precipitously if animals were exposed to heat, direct sunlight and left in seawater-filled buckets, or beached prior to collection. Only fresh live animals were used in this study. The cnidae solution was then centrifuged (400× *g*, 20 min, 4 °C) with dampened braking. Pellets were gently resuspended in 4 °C 1 M trisodium citrate solution and centrifuged again to accomplish two washes in 1 M citrate solution (250× *g*, 20 min, 4 °C). Pellets were then diluted 1:0.5 (*v*:*v*) with ice-cold deionized (18.2 MΩ) water rapidly transferred to a 4 °C chilled French Press (SLM Aminco FA-078 FRENCH Pressure Cell Press 115 V) 20 K pressure cell and subjected to 12,000 psi to rupture the cnidae. The lysate was expelled from the pressure cell at 30 drops/min and rapidly recycled through the instrument to achieve >90% cnidae rupture. The final lysate was centrifuged at 12,000× *g* at 4 °C for 5 min to pellet the collagen capsule cnidae wall and tubule. The viscous supernatant (cnidae content, i.e., venom) was then rapidly aliquoted, in 10–500 µL aliquots, and snap-frozen by dropping microcentrifuge tubes into liquid nitrogen and stored at −80 °C.

### 5.2. Size-Exclusion High-Pressure Liquid Chromatography

Size-based chromatographic fractionation of *C. fleckeri* and *A. alata* crude venom was performed using an analytical Bio-Sil SEC-125 column, 5 µm, 7.8 × 100 mm (BioRad 125-0060 with BioSep-SEC-S 3000 and GFC-3000) equilibrated with 0.5 M ammonium bicarbonate or sodium phosphate buffer (50 mM Na_2_HPO_4_, 50 mM NaHPO_4_, 150 mM NaCl buffer, pH 6.8) using an AKTA Purifier HPLC system (GE Bioscience).

*P. physalis* venom was subjected to size-exclusion chromatography using Superdex 200 HR 10/30, 13 µm, 10 × 300 mm (Amersham) using an AKTA Purifier HPLC system (GE Bioscience). The column was washed with a sodium phosphate buffer (50 mM Na_2_HPO_4_, 50 mM NaHPO_4_, 150 mM NaCl buffer, pH 6.8).

An injection volume of 100 µL of 0.45 micron filtered crude venom at 10 mg/mL was used at a flow rate of 0.5 mL/min for 30 min. The chromatograms were recorded with a triple UV detector at 215 nm, 254 nm, 280 nm, 330 nm, and/or 595 nm (mAU) (Figure 5). The eluate fractions (200 µL) were collected in a 96-well plate and stored at −80 °C until use.

### 5.3. Primary DRG Cell Culture

DRG primary cultures were prepared for the constellation pharmacology experiments, using CD1 background, heterozygous transgenic mice. The transgenic line (Tg(Calca-EGFP)FG104Gsat/Mmucd) was created by the Gensat project and generously provided by the Ginty laboratory. In this mouse strain, transcription and translation of GFP-linked calcitonin gene-related peptide (CGRP) in peptidergic neurons renders them fluorescent, allowing rapid identification in the DRG. Experiments were performed using 40- to 50-day-old mice; DRG from both male and female mice were used.

**Figure 13:**
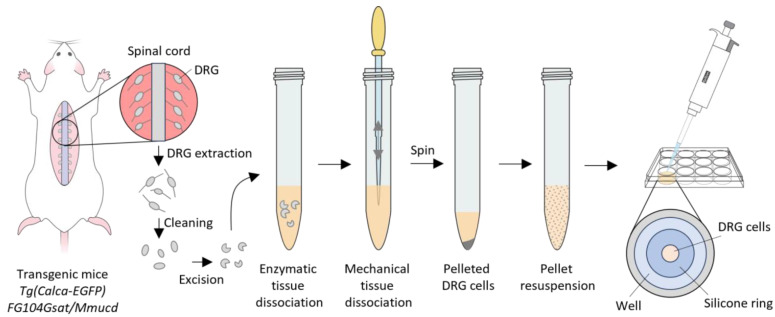
DRG dissection and dissociation protocol. Lumbar DRGs (L1–L6) were removed from the mice and transferred to a petri dish where the nerves were trimmed for cleaning. Individual DRGs were excised to expose the cells. Enzymatic tissue dissociation was performed using a trypsin solution for 20 min at 37 °C. DRGs were then mechanically dissociated by pipetting using Pasteur pipettes with decreasing diameter tips and sieved through a 45-µm cell strainer. Cell suspension was centrifuged and pelleted cells were resuspended in 100 μL of the culture medium. Aliquots of the resuspended solution were plated in 24-well plates within a silicone ring and incubated overnight in a cell culture chamber at 37 °C.

Mice were sacrificed in a CO_2_ chamber and the DRG from the lumbar region (L1–L6) were dissected following previously described methods [21,26,27,32] (Figure 13). Briefly, individual DRG were cleaned by trimming the connecting nerves and then excised to create an aperture for cell exposure. The DRG were then transferred to a 15 mL tube and incubated with 0.1% trypsin for 20 min at 37 °C, vortexing every 5 min. The reaction was quenched by adding three volumes of minimum essential medium (MEM) supplemented with fetal bovine serum (FBS) and PenStrep. The solution was brought to 2 mL, and the DRG cells were mechanically triturated using a sequence of glass pipettes of smaller tip diameters in each step (15–20 times in each step). The solution was then sieved through a 45 µm cell strainer into a new tube. The cell suspension was centrifuged at 14,000 rpm for 10 min using an International Centrifuge (International Equipment Company IEC, Needham Heights, Massachusetts, United States). The cells were resuspended in 100 µL supplemented MEM; 15–20 µL aliquots of the cell suspension were transferred to a poly-*D*-lysine coated, flat-bottom 96-well plate (Corning) within a 2 mm inner diameter silicone ring sitting at the bottom of each well. The cells were allowed to settle for 1 h at 37 °C and then fed with 700 µL of supplemented MEM and incubated overnight at 37 °C before the calcium-imaging experiments.

### 5.4. Constellation Pharmacology

**Figure 14:**
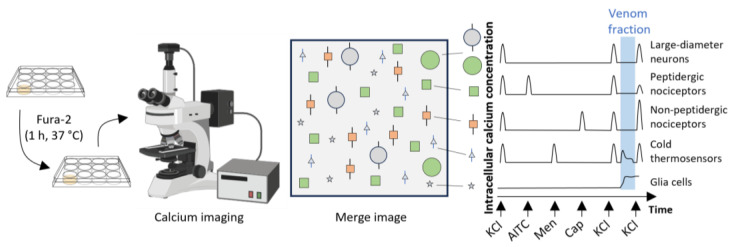
Workflow to screen for effects of cnidarian venom fractions on dorsal root ganglia (DRG) cells using constellation pharmacology. Primary cultures were plated, as described in the Section 5. Eighteen hours later, DRG cells were incubated with Fura-2 for 1 h (30 min in culture chamber and 30 min at room temperature). Continuous Fura-2 calcium imaging was conducted during pulse-chase exposure of the cells in the flow cell well to control solutions, depolarizing KCl pulses and the specific pharmacological agents comprising constellation pharmacology. Venom fractions were applied in between successive KCl pulses (blue box). Cell responses (Fura-2 ratiometric intensity over time) were transformed into profiles of individual cells to be analyzed. KCl, potassium chloride 25 mM; AITC, allyl isothiocyanate (agonist of the transient receptor potential ankyrin 1 channel or TRPA1); Men, menthol (agonist of the transient receptor potential melastatin 8 channel or TRPM8); Cap, capsaicin (agonist of the transient receptor potential vanilloid 1 channel or TRPV1).

The constellation pharmacology assay is summarized in Figure 14. Following overnight incubation, media was replaced with 700 µL of fresh supplemented MEM containing 8.5 μM of Fura-2 (Invitrogen™ eBioscience™ Fura-2 AM Dye) and incubated for 30 min at 37 °C followed by 30 min at room temperature. All the experiments were performed at room temperature. The Fura-2 was removed with multiple washes of “observation solution” (145 mM NaCl, 5 mM KCl, 2 mM CaCl_2_.2H_2_O, 1 mM MgCl_2_.6H_2_O, 10 mM HEPES, 1 mM NaC_6_H_7_O_7_, 10 mM D-glucose, pH 7.4). For intracellular calcium concentration [Ca^2+^]_i_ imaging, we recorded the time-course of the fluorescent emission ratio at 510 nm with alternating excitation wavelengths of calcium bound Fura-2 340 nm and calcium-free Fura-2 380 nm (software allowed live time calculation of the ratio of [(510 nm emission after excitation at 340 nm)/510 nm emission after excitation at 380 nm)]). Thus, changes in the [Ca^2+^]_i_ were followed after a 10 s pulse of each tracer, washing three times after application with a 5 min interval before the following pulse. All agonists were prepared in observation solution; these consisted of 25 and 40 mM KCl, 100 µM allyl isothiocyanate (AITC), 400 µM menthol, 300 nM capsaicin, and 1 µM kM-RIIIJ conotoxin [17,21,46]. Protocols comprising each experiment were completed within 1.5 h. In cnidarian crude venom or fractions tests, aliquots were freshly thawed and diluted in the observation solution. An initial KCl pulse was administered, followed by incubation with the 1X PBS for 3.5 min as a negative control. At 3.5 min, another KCl pulse was administered, followed by the addition of the venom in the observation solution. This was incubated for 3.5 min, followed by a KCl pulse. After the experiments using the crude venom, cells were incubated with 0.4% trypan blue solution (Sigma-Aldrich, St. Louis, MO, USA) diluted with an equal volume of the observation solution for 5 min and imaged under a bright field microscope to quantitate cell viability. After the experiments with the venom fractions, cells were sequentially stained using 0.0025 mg/mL Alexa-Fluor 568 isolectin B4 (IB4, Invitrogen, Waltham, MA, USA) to identify non-peptidergic neurons and 0.027 mg/mL Hoechst 33,342 (Thermo Fisher Scientific, Waltham, MA, USA) to visualize the nuclei. Cell culture images were obtained using NIS-Elements (Nikon Instruments, Melville, NY, USA) software. Images were analyzed using CellProfiler 2.2.0 [87] to define the region of interest (ROI) and to obtain cell parameters and the time course values of the 340:380 ratio for each cell. Quantitative and visual analyses were conducted in R (Version 3.6.3, 29 February 2020, “Holding the Windsock” 2020©, The R Foundation for Statistical Computing Platform: x86_64-w64-mingw32/x64 (64-bit) http://www.R-project.org/ accessed on 16 October 2024), as previously reported [21,26,27].

## Figures and Tables

**Table 1:** Summary of Dorsal Root Ganglia (DRG) cell characteristics and pharmacological responses used for cell classification. DRG were excised from transgenic mice expressing green fluorescent protein (GFP) coupled with the calcitonin gene-related protein (CGRP) to identify peptidergic neurons. Primary culture DRG cells were labeled using fluorescently labeled isolectin B4 (IB4), a glycan-specific lectin used to identify non-peptidergic nociceptors, a specific neuronal subclass. The presence/absence of VGCC in the satellite glial cells, the main glial cell class in the DRG, is rather controversial: although calcium currents (I_Ca_) have been detected in non-neuronal cells from the DRG using whole-cell patch clamp [29], they have not been directly detected by other methods, and it is generally accepted that they lack VGCC [30,31]. The table includes pertinent (but not all) ion channels and receptors found in DRG cells. Legend for table symbols: glia, gray star; large diameter neurons L1-L4, gray circle with lines; large diameter neurons L5-L6, green circle; peptidergic nociceptor medium diameter neurons, green square; non-petidergic nociceptor medium diameter neurons, orange square with lines; small diameter neurons, gray triangle with lines.

Cell Class		Cell Subclass	Voltage Gated Channels				TRP Channels			GFP +	IB4 +
			Ca	Na	K	Cl	V1	A1	M8		
Glia	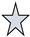	-	+/−	−	+	+	−	−	−	−	−
Large diameter neurons	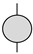	L1–L4	+	+	+	+	−	−	−	−	−
	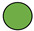	L5–L6	+	+	+	+	−	−	−	+	−
Medium diameter neurons	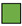	Peptidergic nociceptors	+	+	+	+	+	+	+	+	−
	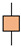	Non-peptidergic nociceptors	+	+	+	+	+	+	−	−	+
Small diameter neurons	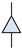	-	+	+	+	+	+	+	+	−	−

## Data Availability

Raw data will be available upon request.

## Supplementary Materials

The following supporting information can be downloaded at: www.mdpi.com/xxx/s1, Table S1: Census of the number of cells responding to *Physalia physalis* venom fraction; Table S2: Census of the number of cells responding to *Chironex fleckeri* venom fraction; Table S3: Census of the number of cells responding to *Alatina alata* venom fraction; Table S4: Census of the number of cells responding to *Alatina alata* venom subfractions.

## References

[1] Yanagihara A., Shohet R.V. (2012). Cubozoan Venom-Induced Cardiovascular Collapse Is Caused by Hyperkalemia and Prevented by Zinc Gluconate in Mice. PLoS ONE.

[2] Yanagihara A., Wilcox C., Smith J., Surrett G. (2016). Cubozoan Envenomations: Clinical Features, Pathophysiology, and Management. The Cnidaria, Past Present and Future.

[3] Jouiaei M., Yanagihara A.A., Madio B., Nevalainen T.J., Alewood P.F., Fry B.G. (2015). Ancient Venom Systems: A Review on Cnidaria Toxins. Toxins.

[4] D’Ambra I., Lauritano C. (2020). A Review of Toxins from Cnidaria. Mar. Drugs.

[5] Tibballs J., Yanagihara A.A., Turner H.C., Winkel K. (2011). Immunological and Toxinological Responses to Jellyfish Stings. Inflamm. Allergy Drug Targets.

[6] Brinkman D.L., Jia X., Potriquet J., Kumar D., Dash D., Kvaskoff D., Mulvenna J. (2015). Transcriptome and Venom Proteome of the Box Jellyfish Chironex Fleckeri. BMC Genom..

[7] Brinkman D.L., Burnell J.N. (2009). Biochemical and Molecular Characterisation of Cubozoan Protein Toxins. Toxicon.

[8] Jouiaei M., Casewell N.R., Yanagihara A.A., Nouwens A., Cribb B.W., Whitehead D., Jackson T.N.W., Ali S.A., Wagstaff S.C., Koludarov I. (2015). Firing the Sting: Chemically Induced Discharge of Cnidae Reveals Novel Proteins and Peptides from Box Jellyfish (*Chironex fleckeri*) Venom. Toxins.

[9] Beress L., Bruhn T., Sanchez-Rodriguez J., Wachter E., Schweitz H., Rochat H., Martin-Eauclaire M. (2000). Sea Anemone Toxins, Action on Na+ Channels and K+ Channels: Isolation and Characterization. Animal Toxins—Facts and Protocols.

[10] Mandrek K., Milenov K. (1991). Responses of Porcine Gastric and Duodenal Smooth Muscle to VIP. J. Auton. Pharmacol..

[11] Wollberg Z., Bdolah A., Galron R., Sokolovsky M., Kochva E. (1991). Contractile Effects and Binding Properties of Endothelins/Sarafotoxins in the Guinea Pig Ileum. Eur. J. Pharmacol..

[12] Fletcher J.E., Adnet P.J., Reyford H., Wieland S.J., Stewart S.L., Rosenberg H. (1999). ATX II, a Sodium Channel Toxin, Sensitizes Skeletal Muscle to Halothane, Caffeine, and Ryanodine. Anesthesiology.

[13] Arkin M.R., Connor P.R., Emkey R., Garbison K.E., Heinz B.A., Wiernicki T.R., Johnston P.A., Kandasamy R.A., Rankl N.B., Sittampalam S. (2004). FLIPRTM Assays for GPCR and Ion Channel Targets. Assay Guidance Manual.

[14] Prashanth J.R., Hasaballah N., Vetter I. (2017). Pharmacological Screening Technologies for Venom Peptide Discovery. Neuropharmacology.

[15] Hille B. (2001). Ion Channels of Excitable Membranes.

[16] Maatuf Y., Priel A., Priel A. (2020). High-Throughput Calcium Imaging Screen of Toxins’ Function in Dissociated Sensory Neurons. Snake and Spider Toxins. Methods in Molecular Biology.

[17] Teichert R.W., Memon T., Aman J.W., Olivera B.M. (2014). Using Constellation Pharmacology to Define Comprehensively a Somatosensory Neuronal Subclass. Proc. Natl. Acad. Sci. USA.

[18] Teichert R.W., Raghuraman S., Memon T., Cox J.L., Foulkes T., Rivier J.E., Olivera B.M. (2012). Characterization of Two Neuronal Subclasses through Constellation Pharmacology. Proc. Natl. Acad. Sci. USA.

[19] Teichert R.W., Schmidt E.W., Olivera B.M. (2015). Constellation Pharmacology: A New Paradigm for Drug Discovery. Annu. Rev. Pharmacol. Toxicol..

[20] Curtice K.J., Leavitt L.S., Chase K., Raghuraman S., Horvath M.P., Olivera B.M., Teichert R.W. (2016). Classifying Neuronal Subclasses of the Cerebellum through Constellation Pharmacology. J. Neurophysiol..

[21] Giacobassi M.J., Leavitt L.S., Raghuraman S., Alluri R. (2020). An Integrative Approach to the Facile Functional Classification of DRG Neuronal Subclasses. Proc. Natl. Acad. Sci. USA.

[22] Raghuraman S., Garcia A.J., Anderson T.M., Twede V.D., Curtice K.J., Chase K., Ramirez J.-M., Olivera B.M., Teichert R.W. (2014). Defining Modulatory Inputs into CNS Neuronal Subclasses by Functional Pharmacological Profiling. Proc. Natl. Acad. Sci. USA.

[23] Inagaki R.T., Raghuraman S., Chase K., Steele T., Zornik E., Olivera B., Yamaguchi A. (2020). Molecular Characterization of Frog Vocal Neurons Using Constellation Pharmacology. J. Neurophysiol..

[24] Raghuraman S., Xie J.Y., Giacobassi M.J., Tun J.O., Chase K., Lu D., Teichert R.W., Porreca F., Olivera B.M. (2020). Chronicling Changes in the Somatosensory Neurons after Peripheral Nerve Injury. Proc. Natl. Acad. Sci. USA.

[25] Imperial J.S., Cabang A.B., Song J., Raghuraman S., Gajewiak J., Watkins M., Showers-Corneli P., Fedosov A., Concepcion G.P., Terlau H. (2014). A Family of Excitatory Peptide Toxins from Venomous Crassispirine Snails: Using Constellation Pharmacology to Assess Bioactivity. Toxicon Off. J. Int. Soc. Toxinology.

[26] Chua V.M., Gajewiak J., Watkins M., Espino S.S., Ramiro I.B.L., Omaga C.A., Imperial J.S., Carpio L.P.D., Fedosov A., Safavi-Hemami H. (2020). Purification and Characterization of the Pink-Floyd Drillipeptide, a Bioactive Venom Peptide from *Clavus davidgilmouri* (Gastropoda: Conoidea: Drilliidae). Toxins.

[27] Bosse G.D., Urcino C., Watkins M., Flórez Salcedo P., Kozel S., Chase K., Cabang A., Espino S.S., Safavi-Hemami H., Raghuraman S. (2021). Discovery of a Potent Conorfamide from *Conus episcopatus* Using a Novel Zebrafish Larvae Assay. J. Nat. Prod..

[28] Neves J.L.B., Urcino C., Chase K., Dowell C., Hone A.J., Morgenstern D., Chua V.M., Ramiro I.B.L., Imperial J.S., Leavitt L.S. (2024). Using Constellation Pharmacology to Characterize a Novel α-Conotoxin from *Conus ateralbus*. Mar. Drugs.

[29] Verkhratsky A., Steinhäuser C. (2000). Ion Channels in Glial Cells. Brain Res. Brain Res. Rev..

[30] Hanani M., Spray D.C. (2020). Emerging Importance of Satellite Glia in Nervous System Function and Dysfunction. Nat. Rev. Neurosci..

[31] Cherkas P.S., Huang T.-Y., Pannicke T., Tal M., Reichenbach A., Hanani M. (2004). The Effects of Axotomy on Neurons and Satellite Glial Cells in Mouse Trigeminal Ganglion. Pain.

[32] Paguigan N., Tun J.O., Leavitt L.S., Chase K., Dowell C., Deering-Rice C.E., Lim A.L., Karthikeyan M., Hughen R.W., Zhang J. (2021). Nicotinic Acetylcholine Receptor Partial Antagonist Polyamides from Tunicates and Their Predatory Sea Slugs. ACS Chem. Neurosci..

[33] Chung J.J., Ratnapala L.A., Cooke I.M., Yanagihara A.A. (2001). Partial Purification and Characterization of a Hemolysin (CAH1) from Hawaiian Box Jellyfish (*Carybdea alata*) Venom. Toxicon.

[34] Flecker H. (1945). Injuries by Unknown Agents to Bathers in North Queensland. Med. J. Aust..

[35] Flecker H. (1952). Fatal Stings to North Queensland Bathers. Med. J. Aust..

[36] Barnes J.H. (1960). Observations on Jellyfish Stingings in North Queensland. Med. J. Aust..

[37] Sonthichai C., Tikumrum S., Smithsuwan P., Bussarawit S., Sermgew T. (2009). Jellyfish Envenomation Events in Selected Coastal Provinces of Thailand 1998-2008. Outbreak Surveill. Investig. Response.

[38] Pirkle C.M., Yanagihara A.A. (2019). Insights in Public Health: Trapped in a Sea of Uncertainty: Limitations in Unintentional Injury Research in the Philippines and Interdisciplinary Solutions to Reduce Fatal Box Jellyfish Stings. Hawaii J. Med. Public Health J. Asia Pac. Med. Public Health.

[39] Lawley J.W., Ames C.L., Bentlage B., Yanagihara A., Goodwill R., Kayal E., Hurwitz K., Collins A.G. (2016). Box Jellyfish *Alatina alata* Has a Circumtropical Distribution. Biol. Bull..

[40] Yanagihara A.A. (2019). Methods and Compositions for Treating and/or Inhibiting Toxins Using Copper-Containing Compounds. U.S. Patent.

[41] Le Gall F., Favreau P., Richard G., Letourneux Y., Molgó J. (1999). The Strategy Used by Some Piscivorous Cone Snails to Capture Their Prey: The Effects of Their Venoms on Vertebrates and on Isolated Neuromuscular Preparations. Toxicon.

[42] Van den Bergh V., Boens N., De Schryver F.C., Ameloot M., Steels P., Gallay J., Vincent M., Kowalczyk A. (1995). Photophysics of the Fluorescent Ca2+ Indicator Fura-2. Biophys. J..

[43] Caterina M.J., Schumacher M.A., Tominaga M., Rosen T.A., Levine J.D., Julius D. (1997). The Capsaicin Receptor: A Heat-Activated Ion Channel in the Pain Pathway. Nature.

[44] Jordt S.-E., Bautista D.M., Chuang H., McKemy D.D., Zygmunt P.M., Högestätt E.D., Meng I.D., Julius D. (2004). Mustard Oils and Cannabinoids Excite Sensory Nerve Fibres through the TRP Channel ANKTM1. Nature.

[45] McKemy D.D., Neuhausser W.M., Julius D. (2002). Identification of a Cold Receptor Reveals a General Role for TRP Channels in Thermosensation. Nature.

[46] Cordeiro S., Finol-Urdaneta R.K., Köpfer D., Markushina A., Song J., French R.J., Kopec W., de Groot B.L., Giacobassi M.J., Leavitt L.S. (2019). Conotoxin κM-RIIIJ, a Tool Targeting Asymmetric Heteromeric Kv1 Channels. Proc. Natl. Acad. Sci. USA.

[47] Honma T., Shiomi K. (2006). Peptide Toxins in Sea Anemones: Structural and Functional Aspects. Mar. Biotechnol..

[48] Honma T., Kawahata S., Ishida M., Nagai H., Nagashima Y., Shiomi K. (2008). Novel Peptide Toxins from the Sea Anemone *Stichodactyla haddoni*. Peptides.

[49] Oliveira J.S., Zaharenko A.J., Ferreira W.A., Konno K., Shida C.S., Richardson M., Lúcio A.D., Beirão P.S.L., de Freitas J.C. (2006). BcIV, a New Paralyzing Peptide Obtained from the Venom of the Sea Anemone Bunodosoma Caissarum. A Comparison with the Na+ Channel Toxin BcIII. Biochim. Biophys. Acta.

[50] Cuypers E., Peigneur S., Debaveye S., Shiomi K., Tytgat J. (2011). TRPV1 Channel as New Target for Marine Toxins: Example of Gigantoxin I, a Sea Anemone Toxin Acting Via Modulation of the PLA2 Pathway. Acta Chim. Slov..

[51] Kasheverov I.E., Logashina Y.A., Kornilov F.D., Lushpa V.A., Maleeva E.E., Korolkova Y.V., Yu J., Zhu X., Zhangsun D., Luo S. (2022). Peptides from the Sea Anemone *Metridium senile* with Modified Inhibitor Cystine Knot (ICK) Fold Inhibit Nicotinic Acetylcholine Receptors. Toxins.

[52] Holstein T., Tardent P. (1984). An ultrahigh-speed analysis of exocytosis: Nematocyst discharge. Science.

[53] Carrette T., Seymour J. (2004). A Rapid and Repeatable Method for Venom Extraction from Cubozoan Nematocysts. Toxicon Off. J. Int. Soc. Toxinology.

[54] Mustafa M.R., White E., Hongo K., Othman I., Orchard C.H. (1995). The Mechanism Underlying the Cardiotoxic Effect of the Toxin from the Jellyfish *Chironex fleckeri*. Toxicol. Appl. Pharmacol..

[55] Bailey P., Bakker A., Seymour J., Wilce J. (2005). A Functional Comparison of the Venom of Three Australian Jellyfish–*Chironex fleckeri*, *Chiropsalmus* sp., and *Carybdea xaymacana*—On Cytosolic Ca^2+^, Haemolysis and *Artemia* sp. Lethality. Toxicon.

[56] Bloom D.A., Burnett J.W., Alderslade P. (1998). Partial Purification of Box Jellyfish (*Chironex fleckeri*) Nematocyst Venom Isolated at the Beachside. Toxicon Off. J. Int. Soc. Toxinology.

[57] Winkel K., Tibballs J., Molenaar P., Lambert G., Coles P., Ross-Smith M., Kabore C., Fenner P., Gershwin L., Hawdon G. (2005). Cardiovascular Actions of the Venom from the Irukandji (*Carukia barnesi*) Jellyfish: Effects in Human, Rat and Guinea-Pig Tissues *In Vitro* and in Pigs *In Vivo*. Clin. Exp. Pharmacol. Physiol..

[58] Lausen B., Ahang A., Cummins S., Wang T. (2023). Investigation of Best Practices for Venom Toxin Purification in Jellyfish towards Functional Characterisation. Toxins.

[59] Li A., Yu H., Li R., Yue Y., Yu C., Geng H., Liu S., Xing R., Li P. (2022). Jellyfish *Nemopilema nomurai* Causes Myotoxicity through the Metalloprotease Component of Venom. Biomed. Pharmacother..

[60] Yu C., Yin X., Li A., Li R., Yu H., Xing R., Liu S., Li P. (2023). Toxin Metalloproteinases Exert a Dominant Influence on Pro-Inflammatory Response and Anti-Inflammatory Regulation in Jellyfish Sting Dermatitis. J. Proteom..

[61] Hwang S.J., Ahn E.-Y., Park Y., Lee H.-J. (2018). An Aqueous Extract of Nomura’s Jellyfish Ameliorates Inflammatory Responses in Lipopolysaccharide-Stimulated RAW264.7 Cells and a Zebrafish Model of Inflammation. Biomed. Pharmacother..

[62] Yu C., Li R., Yin X., Yu H., Li P. (2021). Synergistic Effect of Proteinase Activity by Purification and Identification of Toxic Protease From *Nemopilema nomurai*. Front. Pharmacol..

[63] Burnett J.W., Long K.O., Rubinstein H.M. (1992). Beachside Preparation of Jellyfish Nematocyst Tentacles. Toxicon.

[64] Brinkman D.L., Konstantakopoulos N., McInerney B.V., Mulvenna J., Seymour J.E., Isbister G.K., Hodgson W.C. (2014). *Chironex fleckeri* (Box Jellyfish) Venom Proteins: Expansion of a cnidarian toxin family that elicits variable cytolytic and cardiovascular effects. J. Biol. Chem..

[65] Yu H., Liu X., Dong X., Li C., Xing R., Liu S., Li P. (2005). Insecticidal Activity of Proteinous Venom from Tentacle of Jellyfish *Rhopilema esculentum* Kishinouye. Bioorg. Med. Chem. Lett..

[66] Beress L., Beress R., Wunderer G. (1975). Isolation and characterisation of three polypeptides with neurotoxic activity from *Anemonia sulcata*. FEBS Lett..

[67] Alama-Bermejo G., Holzer A.S. (2021). Advances and Discoveries in Myxozoan Genomics. Trends Parasitol..

[68] Santander M.D., Maronna M.M., Ryan J.F., Andrade S.C.S. (2022). The State of Medusozoa Genomics: Current Evidence and Future Challenges. GigaScience.

[69] Lara A., Simonson B.T., Ryan J.F., Jegla T. (2023). Genome-Scale Analysis Reveals Extensive Diversification of Voltage-Gated K+ Channels in Stem Cnidarians. Genome Biol. Evol..

[70] Choudhary I., Hwang D.H., Lee H., Yoon W.D., Chae J., Han C.H., Yum S., Kang C., Kim E. (2019). Proteomic Analysis of Novel Components of *Nemopilema nomurai* Jellyfish Venom: Deciphering the Mode of Action. Toxins.

[71] Festoff B.W. (1975). Mechanism of Action of Neurotoxins. Ann. Clin. Lab. Sci..

[72] Terlau H., Olivera B.M. (2004). Conus Venoms: A Rich Source of Novel Ion Channel-Targeted Peptides. Physiol. Rev..

[73] Olivera B.M., Miljanich G.P., Ramachandran J., Adams M.E. (1994). Calcium Channel Diversity and Neurotransmitter Release: The ω-Conotoxins and ω-Agatoxins. Annu. Rev. Biochem..

[74] Liu P., Jo S., Bean B.P. (2012). Modulation of Neuronal Sodium Channels by the Sea Anemone Peptide BDS-I. J. Neurophysiol..

[75] Trim S.A., Trim C.M. (2013). Venom: The Sharp End of Pain Therapeutics. Br. J. Pain.

[76] Bohlen C.J., Julius D. (2012). Receptor-Targeting Mechanisms of Pain-Causing Toxins: How Ow?. Toxicon.

[77] Logashina Y.A., Mosharova I.V., Korolkova Y.V., Shelukhina I.V., Dyachenko I.A., Palikov V.A., Palikova Y.A., Murashev A.N., Kozlov S.A., Stensvåg K. (2017). Peptide from Sea Anemone *Metridium senile* Affects Transient Receptor Potential Ankyrin-Repeat 1 (TRPA1) Function and Produces Analgesic Effect. J. Biol. Chem..

[78] Tonello R., Fusi C., Materazzi S., Marone I.M., De Logu R., Benemei S., Gonçalves M.C., Coppi E., Castro-Junior C.J., Gomez M.V. (2017). The peptide Phα1β, from spider venom, acts as a TRPA1 channel antagonist with antinociceptive effects in mice. Br. J. Pharmacol..

[79] Gui J., Liu B., Cao G., Lipchik A.M., Perez M., Dekan Z., Mobli M., Daly N.L., Alewood P.F., Parker L.L. (2014). A Tarantula-Venom Peptide Antagonizes the TRPA1 Nociceptor Ion Channel by Binding to the S1–S4 Gating Domain. Curr. Biol..

[80] Cuypers E., Yanagihara A., Karlsson E., Tytgat J. (2006). Jellyfish and Other Cnidarian Envenomations Cause Pain by Affecting TRPV1 Channels. FEBS Lett..

[81] Robinson S.D., Safavi-Hemami H., Raghuraman S., Imperial J.S., Papenfuss A.T., Teichert R.W., Purcell A.W., Olivera B.M., Norton R.S. (2015). Discovery by Proteogenomics and Characterization of an RF-Amide Neuropeptide from Cone Snail Venom. J. Proteomics.

[82] Tibballs J., Williams D., Sutherland S.K. (1998). The Effects of Antivenom and Verapamil on the Haemodynamic Actions of *Chironex fleckeri* (Box Jellyfish) Venom. Anaesth. Intensive Care.

[83] Ramasamy S., Isbister G.K., Seymour J.E., Hodgson W.C. (2004). The in Vivo Cardiovascular Effects of Box Jellyfish *Chironex fleckeri* Venom in Rats: Efficacy of Pre-Treatment with Antivenom, Verapamil and Magnesium Sulphate. Toxicon.

[84] Clark G.C., Casewell N.R., Elliott C.T., Harvey A.L., Jamieson A.G., Strong P.N., Turner A.D. (2019). Friends or Foes? Emerging Impacts of Biological Toxins. Trends Biochem. Sci..

[85] Lee C.H., Ruben P.C. (2008). Interaction between Voltage-Gated Sodium Channels and the Neurotoxin, Tetrodotoxin. Channels.

[86] Yanagihara A.A., Kuroiwa J.M.Y., Oliver L.M., Chung J.J., Kunkel D.D. (2002). Ultrastructure of a Novel Eurytele Nematocyst of *Carybdea alata* Reynaud (Cubozoa, Cnidaria). Cell Tissue Res..

[87] Lamprecht M.R., Sabatini D.M., Carpenter A.E. (2018). CellProfiler^TM^: Free, Versatile Software for Automated Biological Image Analysis. BioTechniques.

