# Supplementary material for: Elucidation of Medusozoan (Jellyfish) Venom Constituent Activities Using Constellation Pharmacology
Source: Toxins (Basel). 2024 Oct 17;16(10):447. doi: 10.3390/toxins16100447 (PMC11510950; doi:10.3390/toxins16100447)
Supplement: Supplementary file 1 [file toxins-16-00447-s001.zip › toxins-3194676-supplementary.pdf]

Supplementary material: Elucidation of Medusozoan (Jellyfish) Venom Constituent Activities Using Constellation Pharmacology

Supplementary Table S1. Census of the number of cells responding to *Physalia physalis* venom fraction

Cell Class	Cell Subclass	TRP channel response	1 in 100						1 in 10					
			**Amp**	**Inhibition**	**DE**	**DE+Amp**	**DE+inhib**	**No effect**	**Amp**	**Inhibition**	**DE**	**DE+Amp**	**DE+inhib**	**No effect**
Glia	-	$V1^-A1^-M8^-$	-	-	2 (0.9)	-	-	231 (99.1)	-	-	58 (24.9)	-	-	175 (75.1)
Large-diameter neurons	L1-L4	$V1^-A1^-M8^-$	0 (0.0)	2 (4.2)	3 (6.3)	3 (6.3)	1 (2.1)	39 (81.3)	1 (2.1)	9 (18.8)	7 (14.6)	9 (18.8)	9 (18.8)	13 (27.1)
Medium-diameter neurons	L5-L6	$V1^-A1^-M8^-$	0 (0.0)	4 (21.1)	0 (0.0)	0 (0.0)	0 (0.0)	15 (78.9)	1 (5.3)	9 (47.4)	1 (5.3)	1 (5.3)	2 (10.5)	5 (26.3)
	Peptidergic nociceptors	$M8^+$	0 (0.0)	0 (0.0)	0 (0.0)	0 (0.0)	0 (0.0)	5 (100.0)	0 (0.0)	2 (40.0)	1 (20.0)	0 (0.0)	0 (0.0)	2 (40.0)
		$V1^+A1^-$	6 (2.3)	25 (9.5)	3 (1.1)	9 (3.4)	3 (1.1)	217 (82.5)	11 (4.2)	117 (44.5)	21 (8.0)	47 (17.9)	24 (9.1)	43 (16.3)
		$V1^+A1^+$	4 (5.2)	26 (33.8)	5 (6.5)	0 (0.0)	2 (2.6)	40 (51.9)	7 (9.1)	28 (36.4)	5 (6.5)	20 (26.0)	9 (11.7)	8 (10.4)
		$V1^-A1^+$	0 (0.0)	7 (35.0)	2 (10.0)	1 (5.0)	0 (0.0)	10 (50.0)	2 (10.0)	7 (35.0)	0 (0.0)	5 (25.0)	2 (10.0)	4 (20.0)
	Non-peptidergic nociceptor	$V1^+A1^-$	0 (0.0)	0 (0.0)	0 (0.0)	0 (0.0)	0 (0.0)	15 (100.0)	0 (0.0)	8 (53.3)	1 (6.7)	0 (0.0)	0 (0.0)	6 (40.0)
		$V1^+A1^+$	0 (0.0)	0 (0.0)	0 (0.0)	0 (0.0)	0 (0.0)	6 (100.0)	0 (0.0)	2 (33.3)	0 (0.0)	1 (16.7)	0 (0.0)	3 (50.0)
		$V1^-A1^+$	0 (0.0)	21 (30.0)	0 (0.0)	0 (0.0)	4 (5.7)	45 (64.3)	0 (0.0)	53 (75.7)	0 (0.0)	1 (1.4)	14 (20.0)	2 (2.9)
Small-diameter neurons	Cold thermosensors	$M8^+$	0 (0.0)	4 (4.9)	2 (2.4)	0 (0.0)	0 (0.0)	76 (92.7)	2 (2.4)	17 (20.7)	4 (4.9)	2 (2.4)	5 (6.1)	52 (63.4)
	-	$V1^+A1^-$	2 (2.7)	3 (4.1)	3 (4.1)	1 (1.4)	0 (0.0)	65 (87.8)	2 (2.7)	25 (33.8)	7 (9.5)	14 (18.9)	3 (4.1)	23 (31.1)
	-	$V1^-A1^+$	3 (1.0)	9 (2.9)	2 (0.6)	8 (2.6)	0 (0.0)	287 (92.9)	14 (4.5)	45 (14.6)	12 (3.9)	71 (23.0)	6 (1.9)	161 (52.1)
TOTAL			15 (1.2)	101 (8.3)	22 (1.8)	22 (1.8)	10 (0.8)	1051 (86.1)	40 (3.3)	322 (26.4)	117 (9.6)	171 (14.0)	74 (6.1)	497 (40.7)

The numbers in the table indicate the number of cells. The numbers between parentheses indicate the percentage of cells.

Supplementary Table S2. Census of the number of cells responding to *Chironex fleckeri* venom fraction

Cell Class	Cell Subclass	TRP channel response	1 in 100						1 in 10					
			Amp	Inhibition	DE	DE+Amp	DE+inhib	No effect	Amp	Inhibition	DE	DE+Amp	DE+inhib	No effect
Glia	-	$V1^-A1^-M8^-$	-	-	1 (0.1)	-	-	794 (99.9)	-	-	13 (1.7)	-	-	751 (98.3)
Large-diameter neurons	L1-L4	$V1^-A1^-M8^-$	0 (0.0)	14 (53.8)	0 (0.0)	0 (0.0)	1 (3.8)	11 (42.3)	0 (0.0)	22 (57.9)	0 (0.0)	0 (0.0)	0 (0.0)	16 (42.1)
Medium-diameter neurons	L5-L6 Peptidergic nociceptors	$V1^-A1^-M8^-$	0 (0.0)	2 (66.7)	0 (0.0)	0 (0.0)	0 (0.0)	1 (33.3)	0 (0.0)	12 (80.0)	0 (0.0)	0 (0.0)	0 (0.0)	3 (20.0)
		$M8^+$	0 (0.0)	3 (75.0)	0 (0.0)	0 (0.0)	1 (25.0)	0 (0.0)	0 (0.0)	1 (50.0)	0 (0.0)	0 (0.0)	1 (50.0)	0 (0.0)
		$V1^+A1^-$	2 (0.9)	188 (83.9)	2 (0.9)	0 (0.0)	0 (0.0)	32 (14.3)	4 (1.8)	212 (94.6)	0 (0.0)	2 (0.9)	1 (0.4)	5 (2.2)
		$V1^+A1^+$	2 (2.4)	62 (74.7)	0 (0.0)	1 (1.2)	0 (0.0)	18 (21.7)	1 (0.7)	136 (97.8)	0 (0.0)	1 (0.7)	0 (0.0)	1 (0.7)
		$V1^-A1^+$	0 (0.0)	11 (64.7)	0 (0.0)	0 (0.0)	0 (0.0)	6 (35.3)	0 (0.0)	53 (82.8)	0 (0.0)	0 (0.0)	3 (4.7)	8 (12.5)
	Non-peptidergic nociceptor	$V1^+A1^-$	0 (0.0)	7 (63.6)	0 (0.0)	0 (0.0)	0 (0.0)	4 (36.4)	0 (0.0)	21 (91.3)	0 (0.0)	0 (0.0)	1 (4.3)	1 (4.3)
		$V1^+A1^+$	0 (0.0)	1 (50.0)	0 (0.0)	0 (0.0)	0 (0.0)	1 (50.0)	0 (0.0)	23 (92.0)	0 (0.0)	0 (0.0)	1 (4.0)	1 (4.0)
		$V1^-A1^+$	1 (1.6)	53 (82.8)	0 (0.0)	1 (1.6)	0 (0.0)	9 (14.1)	1 (0.5)	187 (87.4)	0 (0.0)	0 (0.0)	2 (0.9)	24 (11.2)
Small-diameter neurons	Cold thermosensors	$M8^+$	0 (0.0)	10 (13.5)	34 (45.9)	1 (1.4)	18 (24.3)	11 (14.9)	22 (25.0)	42 (47.7)	0 (0.0)	12 (13.6)	8 (9.1)	4 (4.5)
	-	$V1^+A1^-$	2 (6.1)	19 (57.6)	1 (3.0)	1 (3.0)	1 (3.0)	9 (27.3)	1 (2.0)	42 (82.4)	0 (0.0)	2 (3.9)	4 (7.8)	2 (3.9)
	-	$V1^-A1^+$	21 (12.0)	9 (5.1)	1 (0.6)	0 (0.0)	0 (0.0)	144 (82.3)	3 (1.4)	51 (23.1)	0 (0.0)	0 (0.0)	3 (1.4)	164 (74.2)
TOTAL			28 (1.9)	379 (25.1)	39 (2.6)	4 (0.3)	21 (1.4)	1040 (68.8)	32 (1.7)	802 (42.9)	13 (0.7)	17 (0.9)	24 (1.3)	980 (52.5)

The numbers in the table indicate the number of cells. The numbers between parentheses indicate the percentage of cells.

Supplementary Table S3. Census of the number of cells responding to *Alatina alata* venom fraction

Cell Class	Cell Subclass	TRP channel response	1 in 500						1 in 50					
			Amp	Inhibition	DE	DE+Amp	DE+inhib	No effect	Amp	Inhibition	DE	DE+Amp	DE+inhib	No effect
Glia	-	$V1^-A1^-M8^-$	-	-	0 (0.0)	-	-	448 (100.0)	-	-	866 (98.7)	-	-	11 (1.3)
Large-diameter neurons	L1-L4	$V1^-A1^-M8^-$	0 (0.0)	0 (0.0)	0 (0.0)	0 (0.0)	0 (0.0)	21 (100.0)	15 (60.0)	0 (0.0)	0 (0.0)	3 (12.0)	0 (0.0)	7 (28.0)
Medium-diameter neurons	L5-L6 Peptidergic nociceptors	$V1^-A1^-M8^-$	0 (0.0)	0 (0.0)	0 (0.0)	0 (0.0)	0 (0.0)	41 (100.0)	8 (66.7)	0 (0.0)	0 (0.0)	0 (0.0)	0 (0.0)	4 (33.3)
		$M8^+$	3 (14.3)	3 (14.3)	0 (0.0)	0 (0.0)	0 (0.0)	15 (71.4)	1 (50.0)	0 (0.0)	0 (0.0)	1 (50.0)	0 (0.0)	0 (0.0)
		$V1^+A1^-$	26 (13.2)	20 (10.2)	0 (0.0)	0 (0.0)	0 (0.0)	151 (76.6)	28 (13.0)	8 (3.7)	3 (1.4)	10 (4.7)	0 (0.0)	166 (77.2)
		$V1^+A1^+$	7 (13.2)	9 (17.0)	1 (1.9)	0 (0.0)	0 (0.0)	36 (67.9)	18 (20.7)	7 (8.0)	1 (1.1)	7 (8.0)	0 (0.0)	54 (62.1)
		$V1^-A1^+$	14 (23.7)	3 (5.1)	0 (0.0)	1 (1.7)	0 (0.0)	41 (69.5)	6 (16.7)	2 (5.6)	4 (11.1)	4 (11.1)	2 (5.6)	18 (50.0)
	Non-peptidergic nociceptor	$V1^+A1^-$	4 (36.4)	1 (9.1)	0 (0.0)	0 (0.0)	0 (0.0)	6 (54.5)	2 (18.2)	0 (0.0)	0 (0.0)	0 (0.0)	0 (0.0)	9 (81.8)
		$V1^+A1^+$	1 (20.0)	1 (20.0)	0 (0.0)	0 (0.0)	0 (0.0)	3 (60.0)	0 (0.0)	1 (10.0)	1 (10.0)	1 (10.0)	0 (0.0)	7 (70.0)
		$V1^-A1^+$	3 (1.9)	9 (5.7)	2 (1.3)	0 (0.0)	0 (0.0)	144 (91.1)	9 (14.3)	5 (7.9)	16 (25.4)	8 (12.7)	10 (15.9)	15 (23.8)
Small-diameter neurons	Cold thermosensors	$M8^+$	6 (6.8)	1 (1.1)	37 (42.0)	3 (3.4)	1 (1.1)	40 (45.5)	17 (38.6)	1 (2.3)	0 (0.0)	10 (22.7)	0 (0.0)	16 (36.4)
	-	$V1^+A1^-$	7 (29.2)	2 (8.3)	1 (4.2)	0 (0.0)	0 (0.0)	14 (58.3)	5 (13.2)	0 (0.0)	2 (5.3)	18 (47.4)	0 (0.0)	13 (34.2)
	-	$V1^-A1^+$	1 (1.0)	2 (2.0)	3 (3.0)	0 (0.0)	0 (0.0)	95 (94.1)	87 (65.9)	0 (0.0)	2 (1.5)	24 (18.2)	0 (0.0)	19 (14.4)
TOTAL			72 (5.9)	51 (4.2)	44 (3.6)	4 (0.3)	1 (0.1)	1055 (86.0)	196 (12.6)	24 (1.5)	895 (57.7)	86 (5.5)	12 (0.8)	339 (21.8)

The numbers in the table indicate the number of cells. The numbers between parentheses indicate the percentage of cells.

Supplementary Table S4. Census of the number of cells responding to *Alatina alata* venom subfractions

Cell Class	Cell Subclass	TRP channel response	Subfraction A						Subfraction B					
			Amp	Inhibition	DE	DE+Amp	DE+inhib	No effect	Amp	Inhibition	DE	DE+Amp	DE+inhib	No effect
Glia	-	$V1^{-}A1^{-}M8^{-}$	-	-	0 (0.0)	-	-	536 (100.0)	-	-	0 (0.0)	-	-	536 (100.0)
Large-diameter neurons	L1-L4	$V1^{-}A1^{-}M8^{-}$	0 (0.0)	0 (0.0)	0 (0.0)	0 (0.0)	0 (0.0)	5 (100.0)	0 (0.0)	0 (0.0)	0 (0.0)	0 (0.0)	0 (0.0)	5 (100.0)
	L5-L6	$V1^{-}A1^{-}M8^{-}$	0 (0.0)	0 (0.0)	0 (0.0)	0 (0.0)	0 (0.0)	2 (100.0)	0 (0.0)	0 (0.0)	0 (0.0)	0 (0.0)	0 (0.0)	2 (100.0)
Medium-diameter neurons	Peptidergic nociceptors	$M8^{+}$	0 (0.0)	1 (33.3)	0 (0.0)	0 (0.0)	0 (0.0)	2 (66.7)	0 (0.0)	0 (0.0)	0 (0.0)	0 (0.0)	0 (0.0)	3 (100.0)
		$V1^{+}A1^{-}$	14 (26.4)	2 (3.8)	0 (0.0)	2 (3.8)	0 (0.0)	35 (66.0)	15 (28.3)	4 (7.5)	0 (0.0)	3 (5.7)	1 (1.9)	30 (56.6)
		$V1^{+}A1^{+}$	17 (19.5)	8 (9.2)	0 (0.0)	3 (3.4)	0 (0.0)	59 (67.8)	17 (19.5)	3 (3.4)	0 (0.0)	1 (1.1)	0 (0.0)	66 (75.9)
		$V1^{-}A1^{+}$	7 (10.0)	4 (5.7)	0 (0.0)	2 (2.9)	0 (0.0)	57 (81.4)	8 (11.4)	2 (2.9)	0 (0.0)	1 (1.4)	0 (0.0)	59 (84.3)
	Non-peptidergic nociceptor	$V1^{+}A1^{-}$	0 (0.0)	1 (25.0)	0 (0.0)	0 (0.0)	0 (0.0)	3 (75.0)	1 (25.0)	0 (0.0)	0 (0.0)	0 (0.0)	0 (0.0)	3 (75.0)
		$V1^{+}A1^{+}$	0 (0.0)	3 (30.0)	0 (0.0)	0 (0.0)	0 (0.0)	7 (70.0)	0 (0.0)	1 (10.0)	0 (0.0)	0 (0.0)	0 (0.0)	9 (90.0)
		$V1^{-}A1^{+}$	13 (7.9)	8 (4.8)	0 (0.0)	1 (0.6)	0 (0.0)	143 (86.7)	9 (5.5)	1 (0.6)	0 (0.0)	1 (0.6)	0 (0.0)	154 (93.3)
Small-diameter neurons	Cold thermosensors	$M8^{+}$	2 (5.6)	8 (22.2)	0 (0.0)	0 (0.0)	0 (0.0)	26 (72.2)	3 (8.3)	7 (19.4)	0 (0.0)	0 (0.0)	0 (0.0)	26 (72.2)
	-	$V1^{+}A1^{-}$	2 (8.3)	6 (25.0)	2 (8.3)	2 (8.3)	1 (4.2)	11 (45.8)	2 (8.3)	3 (12.5)	2 (8.3)	2 (8.3)	1 (4.2)	14 (58.3)
	-	$V1^{-}A1^{+}$	2 (3.6)	3 (5.5)	0 (0.0)	0 (0.0)	0 (0.0)	50 (90.9)	1 (1.8)	4 (7.3)	0 (0.0)	0 (0.0)	0 (0.0)	50 (90.9)
TOTAL			57 (5.4)	44 (4.2)	2 (0.2)	10 (1.0)	1 (0.1)	936 (89.1)	56 (5.3)	25 (2.4)	2 (0.2)	8 (0.8)	2 (0.2)	957 (91.1)

The numbers in the table indicate the number of cells. The numbers between parentheses indicate the percentage of cells.

It continues in the next page.

Supplementary Table S4 *(continuation)*. Census of the number of cells responding to *Alatina alata* venom subfractions

Cell Class	Cell Subclass	TRP channel response	Subfraction C						Subfraction D					
			Amp	Inhibition	DE	DE+Amp	DE+inhib	No effect	Amp	Inhibition	DE	DE+Amp	DE+inhib	No effect
Glia	-	$V1^-A1^-M8^-$	-	-	2 (0.2)	-	-	987 (99.8)	-	-	3 (0.3)	-	-	986 (99.7)
Large-diameter neurons	L1-L4	$V1^-A1^-M8^-$	0 (0.0)	1 (1.6)	0 (0.0)	1 (1.6)	1 (1.6)	58 (95.1)	1 (1.6)	2 (3.3)	2 (3.3)	1 (1.6)	0 (0.0)	55 (90.2)
	L5-L6	$V1^-A1^-M8^-$	0 (0.0)	0 (0.0)	0 (0.0)	0 (0.0)	0 (0.0)	37 (100.0)	2 (5.4)	0 (0.0)	0 (0.0)	0 (0.0)	0 (0.0)	35 (94.6)
Medium-diameter neurons	Peptidergic nociceptors	$M8^+$	0 (0.0)	0 (0.0)	0 (0.0)	0 (0.0)	0 (0.0)	1 (100.0)	0 (0.0)	0 (0.0)	0 (0.0)	0 (0.0)	0 (0.0)	1 (100.0)
		$V1^+A1^-$	5 (1.8)	11 (3.9)	1 (0.4)	1 (0.4)	0 (0.0)	263 (93.6)	4 (1.4)	16 (5.7)	0 (0.0)	0 (0.0)	0 (0.0)	261 (92.9)
		$V1^+A1^+$	1 (2.8)	3 (8.3)	0 (0.0)	0 (0.0)	0 (0.0)	32 (88.9)	2 (5.6)	1 (2.8)	0 (0.0)	0 (0.0)	0 (0.0)	33 (91.7)
		$V1^-A1^+$	3 (6.8)	4 (9.1)	0 (0.0)	0 (0.0)	1 (2.3)	36 (81.8)	1 (2.3)	2 (4.5)	2 (4.5)	1 (2.3)	0 (0.0)	38 (86.4)
	Non-peptidergic nociceptor	$V1^+A1^-$	0 (0.0)	1 (3.1)	0 (0.0)	0 (0.0)	0 (0.0)	31 (96.9)	0 (0.0)	1 (3.1)	0 (0.0)	0 (0.0)	0 (0.0)	31 (96.9)
		$V1^+A1^+$	0 (0.0)	0 (0.0)	0 (0.0)	0 (0.0)	0 (0.0)	1 (100.0)	0 (0.0)	0 (0.0)	0 (0.0)	0 (0.0)	0 (0.0)	1 (100.0)
		$V1^-A1^+$	0 (0.0)	0 (0.0)	1 (1.1)	0 (0.0)	0 (0.0)	90 (98.9)	0 (0.0)	2 (2.2)	0 (0.0)	0 (0.0)	0 (0.0)	89 (97.8)
Small-diameter neurons	Cold thermosensors	$M8^+$	1 (1.3)	1 (1.3)	1 (1.3)	0 (0.0)	0 (0.0)	72 (96.0)	0 (0.0)	4 (5.3)	4 (5.3)	0 (0.0)	0 (0.0)	67 (89.3)
	-	$V1^+A1^-$	1 (2.1)	2 (4.3)	1 (2.1)	0 (0.0)	0 (0.0)	43 (91.5)	1 (2.1)	0 (0.0)	0 (0.0)	0 (0.0)	0 (0.0)	46 (97.9)
	-	$V1^-A1^+$	0 (0.0)	3 (2.2)	0 (0.0)	0 (0.0)	0 (0.0)	132 (97.8)	0 (0.0)	5 (3.7)	0 (0.0)	0 (0.0)	0 (0.0)	130 (96.3)
TOTAL			11 (0.6)	26 (1.4)	6 (0.3)	2 (0.1)	2 (0.1)	1783 (97.4)	11 (0.6)	33 (1.8)	11 (0.6)	2 (0.1)	0 (0.0)	1773 (96.9)

The numbers in the table indicate the number of cells. The numbers between parentheses indicate the percentage of cells.
